# Chemical Discrimination and Aggressiveness via Cuticular Hydrocarbons in a Supercolony-Forming Ant, *Formica yessensis*

**DOI:** 10.1371/journal.pone.0046840

**Published:** 2012-10-24

**Authors:** Midori Kidokoro-Kobayashi, Misako Iwakura, Nao Fujiwara-Tsujii, Shingo Fujiwara, Midori Sakura, Hironori Sakamoto, Seigo Higashi, Abraham Hefetz, Mamiko Ozaki

**Affiliations:** 1 Department of Biology, Graduate School of Science, Kobe University, Kobe, Japan; 2 Course in Animal Ecology, Graduate School of Environmental Science, Hokkaido University, Hokkaido, Japan; 3 Venture Laboratory, Kyoto Institute of Technology, Kyoto, Japan; 4 Department of Zoology, George S. Wise Faculty of Life Sciences, Tel Aviv University, Israel; Sheffield University, United States of America

## Abstract

**Background:**

Territorial boundaries between conspecific social insect colonies are maintained through nestmate recognition systems. However, in supercolony-forming ants, which have developed an extraordinary social organization style known as unicoloniality, a single supercolony extends across large geographic distance. The underlying mechanism is considered to involve less frequent occurrence of intraspecific aggressive behaviors, while maintaining interspecific competition. Thus, we examined whether the supercolony-forming species, *Formica yessensis* has a nestmate recognition system similar to that of the multicolonial species, *Camponotus japonicus* with respect to the cuticular hydrocarbon-sensitive sensillum (CHC sensillum), which responds only to non-nestmate CHCs. We further investigated whether the sensory system reflects on the apparent reduced aggression between non-nestmates typical to unicolonial species.

**Methodology/Principal Findings:**

*F. yessensis* constructs supercolonies comprising numerous nests and constitutes the largest supercolonies in Japan. We compared the within-colony or between-colonies’ (1) similarity in CHC profiles, the nestmate recognition cues, (2) levels of the CHC sensillar response, (3) levels of aggression between workers, as correlated with geographic distances between nests, and (4) their genetic relatedness. Workers from nests within the supercolony revealed a greater similarity of CHC profiles compared to workers from colonies outside it. Total response of the active CHC sensilla stimulated with conspecific alien CHCs did not increase as much as in case of *C. japonicus*, suggesting that discrimination of conspecific workers at the peripheral system is limited. It was particularly limited among workers within a supercolony, but was fully expressed for allospecific workers.

**Conclusions/Significance:**

We demonstrate that chemical discrimination between nestmates and non-nestmates in *F. yessensis* was not clear cut, probably because this species has only subtle intraspecific differences in the CHC pattern that typify within a supercolony. Such an incomplete chemical discrimination via the CHC sensilla is thus an important factor contributing to decreased occurrence of intraspecific aggressive behavior especially within a supercolony.

## Introduction

Most ant species and other social organisms rely on recognition systems for either accepting nestmates or aggressively rejecting non-nestmates [1]–[5]. In contrast, unicolonial ant species are typified by constructing supercolonies with no obvious boundaries between nests and permissively accepting workers from any nest within the supercolony [1], [2], [6]. Unicolonial populations of the Argentine ant *Linepithema humile* [7]–[10] and the little fire ant *Wasmannia auropunctata* [11]–[14], for example, construct huge supercolonies in various countries. Unicoloniality thus seems to be a key factor in achieving ecological domination in newly invaded habitats [6]. Genetically, unicoloniality has been hypothesized to occur either as a consequence of a genetic bottleneck [8] or the loss of genetic diversity [9]. Chemically, the importance of similarity in cuticular hydrocarbon (CHC) profiles has been discussed concerning unicoloniality [15], [16].

Unicoloniality is not limited to invasive species, but under certain circumstances can also occur in native ant species populations [17]–[22]. It is assumed that workers of unicolonial species, whether an invasive or a native species, show less frequent severe aggressive behavior toward conspecific individuals but, rather, accept most of them amicably. Nevertheless, workers can still be highly aggressive when they encounter ants that are genetically different and/or that possess different CHC profiles, such as conspecific members of a different supercolony.

*Formica yessensis*, a native Japanese unicolonial species, in the “Ishikari supercolony”, when reported in 1971, consisted of ∼45,000 nests with the total numbers of ∼306,000,000 workers and ∼1,080,000 queens [17]. In 1980s, there was a single event of drastic decrease in the number of nests, but it has gradually recovered. During the active season from spring through autumn, the single supercolony extends across extremely large geographic distance along the Ishikari coast, within which workers and queens frequently migrate among neighboring nests. During wintertime, however, they congregate in a reduced number of nests for hibernation, abandoning other nests.

Behavioral experiments with various ant species revealed both indirectly and directly that aggressive behavior exhibited by workers toward alien ants is elicited by chemosensory detection of differences in chemical signals encoded in CHCs [3], [4], [15], [16], [23]. Despite this well-defined behavior, a sensory organ responsible for nestmate *versus* non-nestmate discrimination was only recently discovered in *Camponotus japonicus* [4]. However, to the best of our knowledge no studies have focused on the chemosensory aspect of aggressive behavior in unicolonial species.

When workers and queens of *F. yessensis*, migrate among neighboring nests in the “Ishikari supercolony”, they are predicted to be less aggressive toward non-nestmates within the supercolony. Unicoloniality allows a single supercolony to extend across extremely large geographic distances, exhibiting lower frequencies of severe aggressive behavior. If unicoloniality of *F*. *yessensis* is also based on similarity/disparity of CHC profiles, some sensory mechanism to detect such differences should be involved in the expression of individual aggressive behavior.

Hence in the present study using the unicolonial *F. yessensis*, we analyzed the CHC profiles and examined the responsiveness of the CHC sensillum, concomitant with recording workers’ aggressive behavior toward conspecific workers within and between supercolonies. We further studied the sensory response and accompanying behaviors also toward *C. japonicus* workers as an interspecific positive control. In the electrophysiological experiments, we investigated a particular type of antennal chemosensilla that responded to CHCs, as a putative sensory organ responsible for conspecific worker discrimination within or between supercolonies and intraspecific worker discrimination. Nonetheless, we cannot exclude additional or alternative contribution of unknown types of antennal chemosensilla. We further considered whether genetic relatedness or similarity/disparity of CHC profile affects the frequency of aggressive behavior occurrence in *F. yessensis*.

## Results

### Similarity of CHC Profiles in a Supercolony

**Figure 1:**
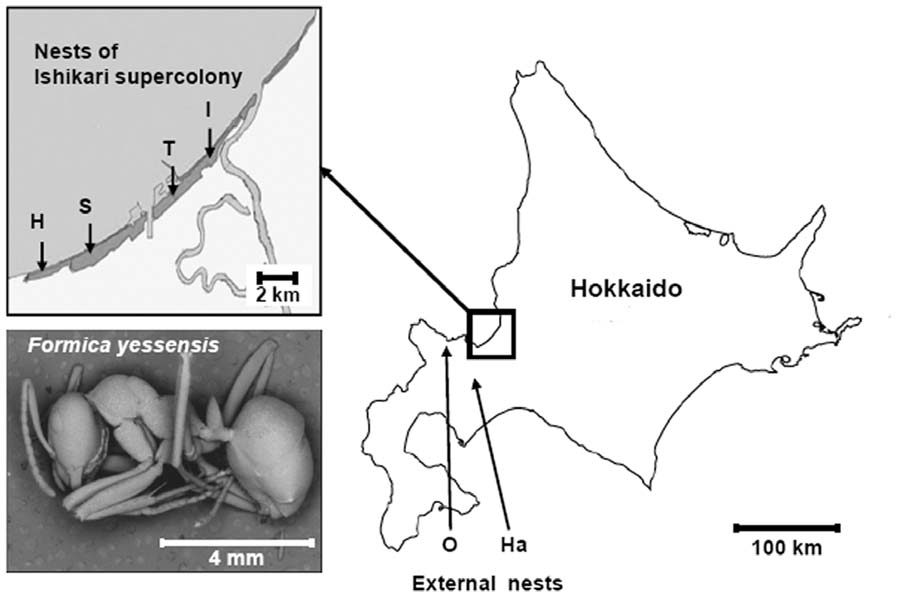
Sampling localities of *F. yessensis*. Location of the “Ishikari supercolony” comprising 4 nests: “Hoshioki” (H); “Shinkawa” (S); “Tarukawa” (T); “Ishikari” (I), and the 2 independent nests, “Oshoro” (O); “Hakkenzan” (Ha), outside the “Ishikari supercolony”. The same letters are used to indicate these nests in Figures 2, 5 and 8. Inset is a scanning electron micrograph (SEM) of a worker of *F. yessensis*.

**Figure 2:**
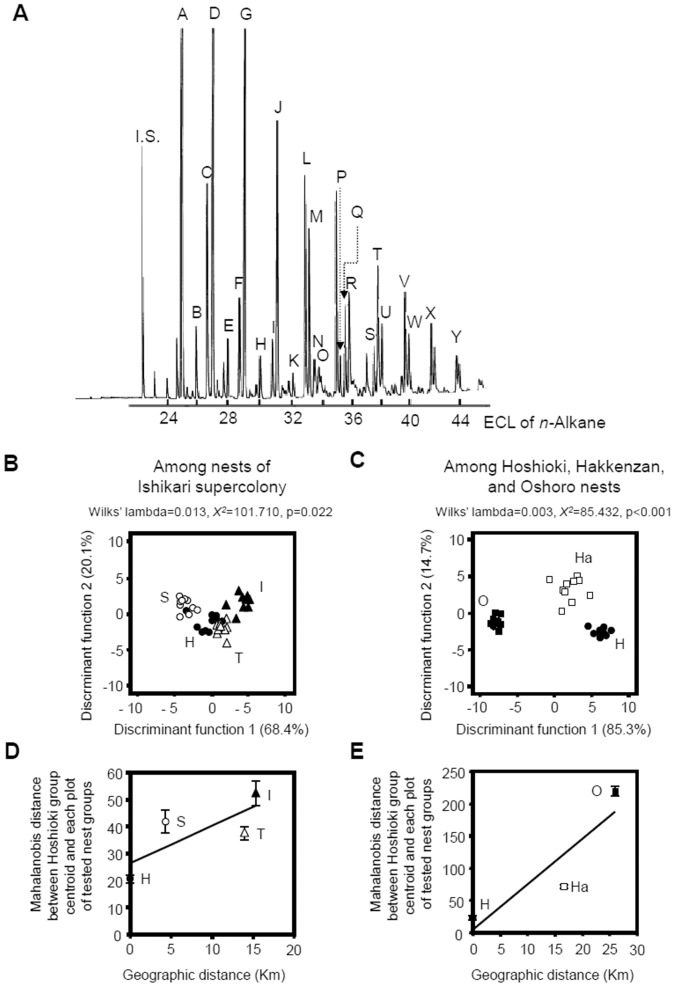
Characteristics of CHC composition of *F. yessensis*. (**A**) Gas-chromatogram of CHCs of *F. yessensis*. (**B**) Discriminant analyses of individual CHC profiles among four nests within the “Ishikari supercolony”; (**C**) Discriminant analyses of individual CHC profiles among three independent nests: “Hoshioki” (H) within and “Oshoro” (O) and “Hakkenzan” (Ha) outside the “Ishikari supercolony”. (**D**) Correlation between geographic distance and the Mahalanobis distance (average±SE) between the 4 nests within the “Ishikari supercolony”; (**E**) Correlation between geographic distance and the Mahalanobis distance (average±SE) between the three independent nests. For the discriminant analyses in (B) and (C) we used 24 peaks (A-X) that were commonly detected in all tested individuals, but omitted the 25th compound, Y, having a too high molecular mass to be chemically identified (see Table 1). The straight lines in (D) and (E) are regression lines drawn by the least-squares method.

We sampled *F. yessensis* workers from four nests (“Hoshioki”, “Shinkawa”, “Tarukawa”, and “Ishikari”) located within the 10-km-wide “Ishikari supercolony” along the Ishikari coast (Figure 1). In addition we sampled two other nests (“Hakkenzan” and “Oshoro”) that are located outside the “Ishikari supercolony”, about 17 and 26 km away from the nearest supercolony boundaries, respectively (Figure 1) [18]. Chemical analysis of worker CHCs revealed blends comprising over 30 compounds, ranging from 25–43 equivalent chain lengths, which were detected in every tested individual regardless of nest. Some peaks contained a mixture of two inseparable branched hydrocarbons. Using 24 structurally identified compounds (A-X in Figure 2A, Table 1), we compared by discriminant analyses the CHC profiles of workers sampled from the four nests within the “Ishikari supercolony” (Figure 2B), and from three independent nests: “Hoshioki”, which belongs to the “Ishikari supercolony”, and “Hakkenzan” and “Oshoro”, both of which are outside the “Ishikari supercolony” (Figure 2C). As depicted in Figure 2B, although ants from each nest clustered together, the nests were not well separated. In contrast, a clear separation among the three independent nests can be seen in Figure 2C. Repeated analyses while replacing the “Hoshioki” nest with each of the three other nests of the supercolony, “Shinkawa”, “Tarukawa”, and “Ishikari”, revealed a similar clear separation between independent nests (Figure S1A”).

**Table 1:** Cutucular hydrocarbon components in *Formica yessensis.*

Peak	Compound
A	*n*-Pentacosane
B	*n*-Hexacosane
C	Heptacosene
D	*n*-Heptacosane
E	*n*-Octacosane
F	Nonacosene
G	*n*-Nonaconane
H	*n*-Triacontane
I	Hentriacontene
J	*n*-Hentriacontane
K	*n*-Dotriacontane
L	Tritriacontene
M	*n*-Tritriacontane
N	11-Methyltritriacontane
O	3-Methyltritriacontane
P	*n*-Pentatriacontene
Q	11-Methylpentatriacontane and 13-Methylpentatriacontane
R	11,12-Dimethylpentatriacontane
S	11-Methylheptatriacontane and 13-Methylheptatriacontane
T	11, X-Dimethylheptatriacontane (X = 12,14,16)
U	Trimethylheptatriacontane
V	13,X-Dimethylnonatriacontane (X = 16,18)
W	Trimethylnonatriacontane
X	13,18-Dimethylhentetracontane
Y	Unidentified

A–Z correspond to the peaks in Figure 2A.

We further assessed whether the disparity in CHC profiles is related to the geographic distances between nests. The Mahalanobis distance is a very useful indicator in multivariate, chemometrical analyses for measuring distances between objects [24], including an estimation of variation in CHC profiles. In accordance with Ugelvig et al. [25], we calculated the Mahalanobis distances between each nest centroid and each individual within the same nest (within-nest variation) and individuals of all other nests within (Figure S2A) or outside of the “Ishikari supercolony” (Figure S2B) (between-nest variation), based on the discriminant analyses in Figures 2B and 2C. Plots of the Mahalanobis distances against the geographic distances between nests are presented in Figures 2D and 2E. Both correlations including the nests within the “Ishikari supercolony” (r = 0.70 at p<0.001 in Figure 2D) and including the independent nests were positive and significant (r = 0.94 at p<0.001 in Figure 2E). Similar positive and significant correlations were found when we substituted the “Hoshioki” nest either with the “Shinkawa” (r = 0.90 at p = 0.018 in Figure S1B left), “Tarukawa” (r = 1.00 at p<0.001 in Figure S1B middle) or “Ishikari” nest (r = 1.00 at p<0.001 in Figure S1B right). We also attempted Mantel test instead of simple correlation tests and found that r = 0.56 at P<0.001 for Figures 2D, r = 0.90 at P<0.001 for Figures 2E, and r = 0.88 at P = 0.018, r = 0.95 at P<0.001 and r = 0.98 at P<0.001 for Figure S1B right, middle and left plates, respectively.

### Responsiveness of the CHC Sensillum to CHCs of Workers from Inside and Outside the Supercolony

**Figure 3:**
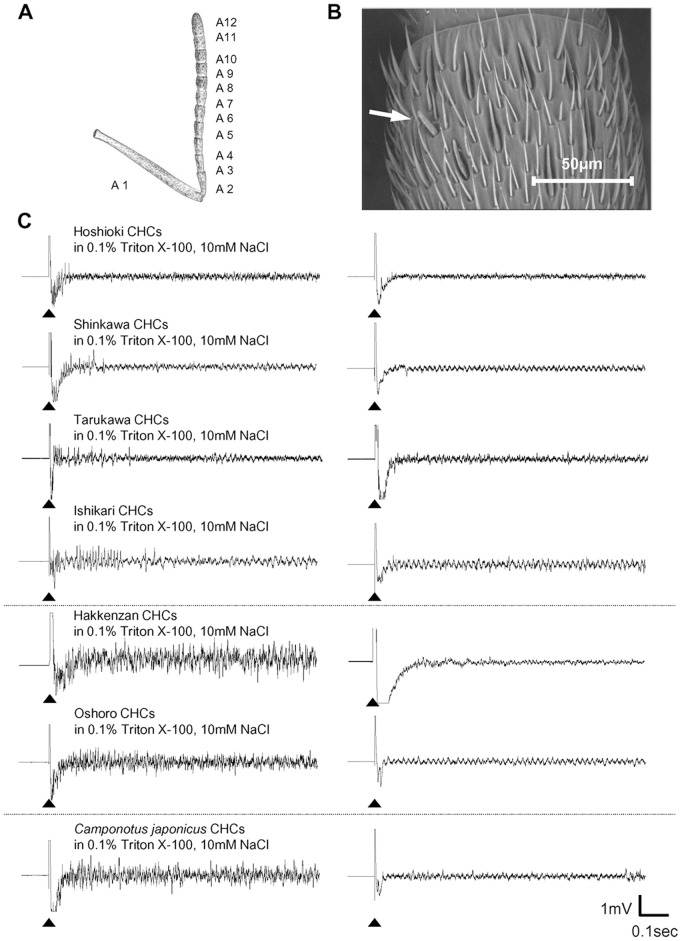
Electrophysiological responses from the CHC sensilla. (**A**) Schematic drawing of an antenna of *F. yessensis*; A1, scape; A2–12, funiculus segments. (**B**) Scanning electron micrograph (SEM) of the antennal surface of A7. A CHC sensillum is indicated by an arrow. (**C**) Left: Representative electrophysiological responses from CHC sensilla of “Hoshioki” workers, when stimulated with CHCs extracted from workers of nests within and outside the “Ishikari supercolony” of *F. yessensis* and workers of *C. japonicus* (Stimulus CHCs were dissolved in 10 mM NaCl plus 0.1% Triton X-100). Right: Control recordings of the same sensilla, when stimulated with the solvent, 10 mM NaCl plus 0.1% Triton X-100. Arrowheads indicate beginning of stimulation.

**Figure 4:**
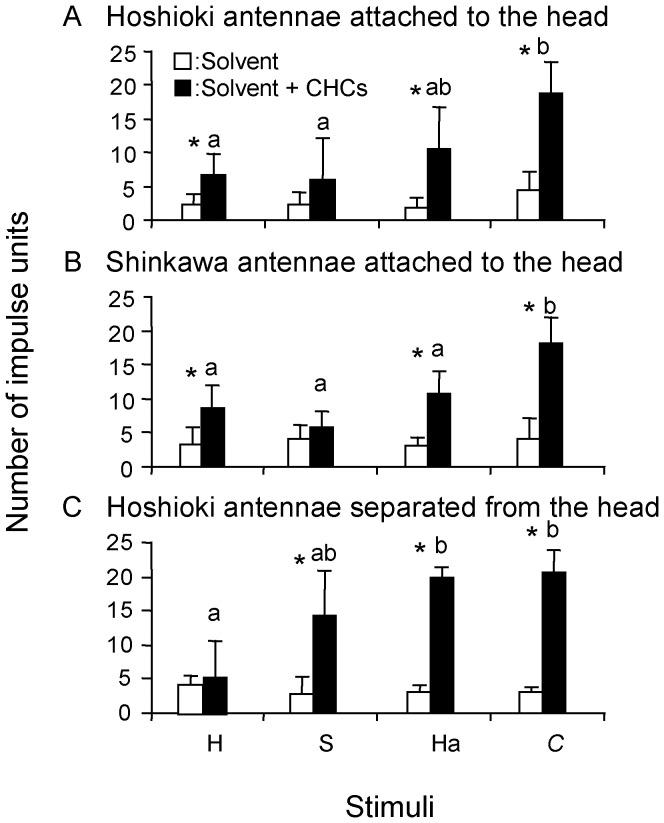
Blind experiments for impulse unit counting. Number of impulse units recruited by the stimulation with “Hoshioki” (H), “Shinkawa” (S), “Hakkenzan” (H) or *C. japonicus* CHCs (*C*) in the three types of CHC sensillar preparations; (**A**) “Hoshioki” CHC sensilla on the antenna attached to the heads; (**B**) “Shinkawa” CHC sensilla on the antenna attached to the heads; (**C**) “Hoshioki” CHC sensilla on the antenna detached from the head. white columns describe the response to the control solvent, 10 mM NaCl plus 0.1% Triton X-100. Different letters indicate significant differences by Tukey HSD test, respectively in plates A–C (A, F = 7.55; B, F = 12.34; C, F = 9.60 at p<0.05). Asterisks indicate significant differences from the control levels by t-test (p<0.05).

Scanning electron microscopy (SEM) of worker *F. yessensis* antenna, comprising 11 funiculus segments and a scape (Figure 3A), revealed morphologically similar sensilla to that of the CHC sensilla originally described in *C. japonicus* [4] (Figure 3B). Each antenna possessed as many as 120 of this type of sensilla (Table S1). These were targeted for the electrophysiological recordings, using CHC blends of the nests of interest. Figure 3C shows a representative set of records from the “Hoshioki” CHC sensilla when stimulated with the CHC blends representing *F. yessensis* workers of the four nests (“Hoshioki”, “Shinkawa”, “Tarukawa”, “Ishikari”) within the “Ishikari supercolony” and the “independent nests” (“Hakkenzan”, “Oshoro”), respectively, or with the CHC blend of workers of *C. japonicus* (which has a markedly different CHC composition [4]). The CHC sensillum of the “Hoshioki” workers occasionally responded even when stimulated with nestmate CHCs, but in most cases the impulses rapidly diminished. Similarly, these CHC sensilla showed sparse type impulses when stimulated with the CHCs of non-nestmates from the other nests within the “Ishikari supercolony”. On the other hand, these CHC sensilla often showed vigorous impulse responses, when stimulated with CHCs of workers from either the “Hakkenzan” or “Oshoro” nests, both of which are outside the “Ishikari supercolony”, and when stimulated with the allospecific CHC blend of *C. japonicus*. The above recordings were not done in a blind manner and each followed the same sequence; the first stimulus constituted the solvent, 10 mM NaCl with 0.1% Triton X-100, and the second the appropriate CHC dissolved in the above solvent. Each sensillum was used only once. A second set of recordings was carried out using the CHC sensilla of either “Hoshioki” or “Shinkawa” workers, which were stimulated with CHCs (dissolved in 10 mM NaCl with 0.1% Triton X-100) of “Hoshioki”, “Shinkawa”, “Hakkenzan”, or *C. japonicus* (Figure 4). The samples were applied in a random and blind manner, with a solvent run preceding each sample. In order to characterize the chemosensory responses of activated receptor neurons in a tested sensillum, we applied a particular program-operating-condition to sort impulse units on the basis of their different shapes. We took no account of data in which less than 9 impulses were generated with the solvent control during 10 s, regardless of their types of impulse unit (see Figure S3). Each receptor neuron generates a particular shape of impulses belonging to a single impulse unit, so that the number of impulse units allows us to estimate how many receptor neurons are involved in the response to multiple CHC components. Thus, the CHC sensilla of “Hoshioki” workers generated 6.80±3.11 and 6.00±6.04 impulse units (average±SD, n = 5) in response to stimuli with “Hoshioki” and “Shinkawa” CHCs, respectively (Figure 4A). The former was significantly different from the control, 2.20±1.79 impulse units, (t = −2.86 at p = 0.02, t-test), while the latter was not significantly different from the control, 2.20±1.92 impulse units, (t = −1.34 at p = 0.22, t-test). In contrast, their response to “Hakkenzan” and *C. japonicus* CHCs recruited 10.60±6.11 (n = 5) and 18.80±4.54 impulse units (n = 6) being significantly higher than their respective controls, 1.80±1.64 impulse units (n = 5) (t = −3.11 at p = 0.014, t-test) and 4.50±2.81 (n = 6) (t = −6.58 at p<0.01, t-test), respectively. Likewise, the responses of the CHC sensilla of “Shinkawa” workers generated 8.80±3.27 and 5.80±2.39 impulse units to stimuli with “Hoshioki” and “Shinkawa” CHCs, respectively (n = 5), but 10.60±3.36 and 18.00±4.00 impulse units to stimuli with “Hakkenzan” (n = 5) and *C. japonicus* CHCs (n = 5), respectively (Figure 4B). In the recordings from the “Hoshioki” (Figure 4A) and “Shinkawa” antennae (Figure 4B), there were no significant differences between numbers of impulse units recruited by the stimuli with CHCs of nestmates and “Hakkenzan” (p>0.05). In contrast, those recruited by the stimuli with CHCs of nestmates and *C. japonicus* were significantly different from each other (p<0.01) (F = 7.55 in Figure 4A; F = 12.34 in Figure 4B, Tukey HSD).

We also tested the CHC sensilla on the isolated antennae that were detached from the head. As shown in Figures 4C, we found that the responding sensilla of “Hoshioki generated 5.25±5.38 and 14.25±6.60 impulse units to stimuli with “Hoshioki” and “Shinkawa” CHCs, respectively (n = 4). The former was not significantly different from the control having 4.25±1.26 impulse units (n = 4) (t = −0.36 at p = 0.73, t-test), while the latter was significantly different from the control having 2.75±2.50 impulse units (n = 4) (t = −3.26 at p = 0.02, t-test). In their response to stimuli with “Hakkenzan” and *C. japonicus* CHCs, we found 20.00±1.41 and 20.75±3.30 impulse units, which were significantly higher than the control, 3.00±1.15 (t = −18.62 at p<0.01, t-test) and 3.00±0.82 impulse units (t = −10.43 at p<0.01, t-test), respectively. There was a significant difference between the number of impulse units recruited by stimulation with CHCs of nestmates and “Hakkenzan” (p<0.05) or *C. japonicus* (p<0.01) (F = 9.60, Tukey HSD) (in Figure 4C). Figure S3 shows the Raster plots corresponding to Figure 4.

**Figure 5:**
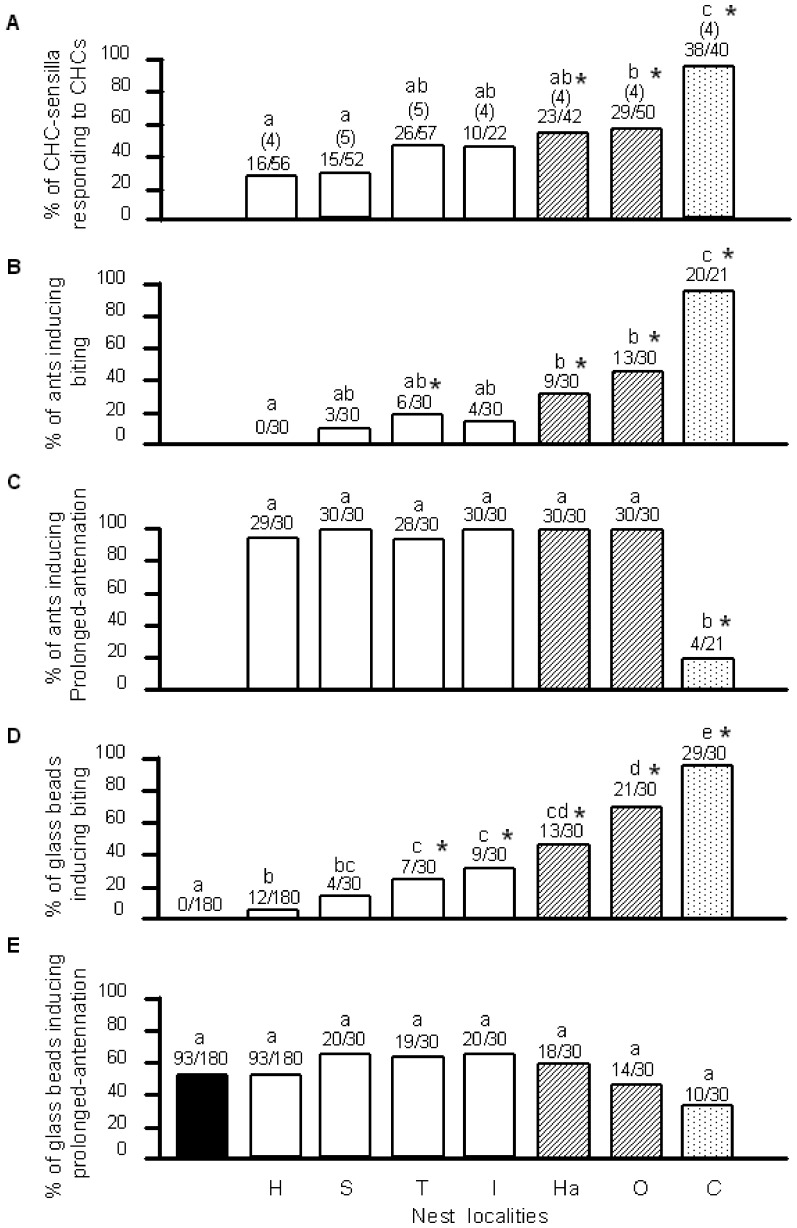
Ratios of activated sensilla and behavioral responses of “Hoshioki” workers to live or surrogate ants. (**A**) Percentage of CHC sensilla in “Hoshioki” workers showing an electrophysiological response to CHCs of workers of *F. yessensis* from nests within (white columns) and outside (hatched columns) the “Ishikari supercolony”, and *C. japonicus* CHCs (dotted columns). The fractions indicate the number of sensilla responding to the stimulus CHCs per total number of sensilla tested. The numbers in parentheses indicate the number of ants used. (**B**) Percentage of workers from the above nests (out of the total workers tested, respectively) that induced biting behavior of “Hoshioki” workers. (**C**) Percentage of workers from the above nests (out of the total workers tested, respectively) that induced prolonged-antennation behavior of “Hoshioki” workers. (**D**) and (**E**) are same as (B) and (C) but using surrogate invader ants made of glass beads coated with purified CHC. Black columns describe the response to *n*-hexane control. Different letters indicate significant differences by Tukey WSD test, respectively in plates A–E. Asterisks indicate significant differences from the control levels responding to “Hoshioki” workers or their CHCs by one-sided Fisher’s exact test with Bonferroni-Holm correction.

During the electrophysiological experiments, we frequently found that a CHC sensillum that responded to CHCs of *C. japonicus* did not always respond to the CHCs of *F. yessensis*. It appears that not only responsiveness of each responding sensillum may contribute to the total chemosensory cue eliciting aggressive behavior, but also the ratio of the responding sensilla. Hence, Figure 5A presents the ratios of responding CHC sensilla of the “Hoshioki” workers, regardless of the number of impulses or impulse units in each recording. Whether the tested sensillum was generating impulses or not during a 10 s recording was first judged by eye and later confirmed in a computer program-operating manner, using SpikeTaro (Chinou Jouhou Shisutemu Inc., Kyoto, Japan) that can check whether the counted impulse number was above or below the control levels. Different letters in Figures 5A–5E indicate significant differences using Tukey WSD test, respectively. A maximum of 46% of the CHC sensilla generated impulses to the CHC blends of workers from the four nests within the “Ishikari supercolony”, whereas 48%, 58%, and 95% of the sensilla responded to the CHC blends of workers from the “Hakkenzan” and “Oshoro” nests and *C. japonicas* workers, respectively. The ratio of sensilla that responded to CHC blends of “Oshoro” and *C. japonicus* workers was significantly higher than the ratio of sensilla that responded to “Hoshioki” CHCs, while the ratio of sensilla that responded to CHCs of “Hakkenzan” was not.

Using one-sided Fisher’s exact test with Bonferroni-Holm correction, revealed that the ratio of “Hoshioki” workers’ sensilla that responded to CHC blend of “Hakkenzan” at p = 0.008<0.013, “Oshoro” at p = 0.002<0.010 or *C. japonicus* at p = 1.08×10^−11^<0.008 was significantly higher than the ratio of sensilla that responded to “Hoshioki” CHCs as a control (indicated with asterisks in Figure 5A–5E). When using the Bonferroni-Holm correction, the rejection region of p values for the one-side Fisher’s exact test was differently defined as <0.013, <0.010 or <0.008 in those cases.

### Aggressive Behavior of Ants from Inside and Outside of a Supercolony

Figures 5B and 5C present, respectively, data from field observations at a “Hoshioki” nest site, pertaining to the elicited aggression (percentage of invading ants that elicited biting) and investigation (percentage of invading ants that elicited >2s of prolonged antennation). Encounters with nestmates were never aggressive, and those with workers from any of the nests belonging to the “Ishikari supercolony” culminated in aggression in less than 20% of the cases, which was not different from that toward “Hoshioki” nestmates. In contrast, in encounters with workers from either of the two independent *F. yessensis* colonies or *C. japonicus* workers, “Hoshioki” workers were significantly more aggressive toward 30% of workers from “Hakkenzan”, 43% of workers from “Oshoro”, and 95% of *C. japonicus* workers. However, a one-sided Fisher’s exact test with Bonferroni-Holm correction (see asterisks in Figure 5B) revealed a significant increase in aggression exhibited by “Hoshioki” workers toward “Tarukawa” workers (p = 0.012<0.017).

The results of the prolonged-antennation presented in Figure 5C show equal investigation for all intraspecific interaction, but much reduced prolonged-antennation frequencies in encounters between *F. yessensis* and *C. japonicus* workers (p = 6.48×10^−9^<0.008, one-sided Fisher's exact test with Bonferroni-Holm correction).

To determine whether the CHCs are the major cues eliciting aggression or prolonged antennation, we repeated the experiment while exposing the “Hoshioki” workers to glass beads coated with purified CHC extracts from workers of the different nests (Figures 5D and 5E). The reaction of the “Hoshioki” workers exhibiting biting toward the CHC-coating glass beads used as surrogate ants was similar to that toward live ants, showing a significant effect (Figure 5D). The correlation coefficient between the data on biting induced by the surrogate and live ants was r = 0.96 at p<0.01 (Figure S5A). Aggression toward the encountered live or surrogate ants was significantly correlated with the Mahalanobis distances (r = 0.89 at p<0.001 in Figure S5B; r = 0.93 at p<0.001 in Figure S5C). As in the case of using live ants, aggression toward surrogate ants with CHCs from either *F. yessensis* workers from independent colonies of “Ishikari supercolony”, “Hakkenzan” and “Oshoro”, or *C. japonicus* workers was significantly higher than the control with nestmate CHCs (Figure 5D). However, one-sided Fisher’s exact test with Bonferroni-Holm correction revealed also significant increases in aggression of “Hoshioki” workers toward “Tarukawa” at p = 0.009<0.017, “Ishikari” at p = 0.001<0.013 from the supercolony, as well as toward “Hakkenzan” at p = 1.31×10^−6^<0.01, “Oshoro” at p = 7.03×10^−14^<0.008 and *C. japonicus* workers at p = 6.74×10^−25^<0.007 (see asterisks in Figure 5D).

As depicted in Figure 5E, there was no significant difference in the prolonged-antennation levels among the various CHC-coating glass beads when using either Tukey WSD test or one-sided Fisher’s exact test with Bonferroni-Holm correction (with hexane, p = 0.542>0.01; “Shinkawa” CHCs, p = 0.959>0.025; “Tarukawa” CHCs, p = 0.918>0.017; “Ishikari” CHCs, p = 0.959>0.050 “Hakkenzan” CHCs, p = 0.852>0.013; “Oshoro” CHCs, p = 0.378>0.008; *C. japonicus* CHCs, p = 0.047>0.007).

The field assay data, using the “Hoshioki” workers as residents (Figures 5B, 5C), were supplemented by blind laboratory tests using either “Hoshioki” (Figure S4A top) or “Shinkawa” workers as residents (Figure S4A bottom). Similar to the field studies, the invader ants had a significant effect on induction of aggressive behavior in the encounters (Tukey WSD test). The resident workers were completely tolerant of both nestmate and supercolonymate workers, but were significantly more aggressive to the allospecific *C. japonicus* (Figure S4A top and bottom). The frequency distribution of prolonged-antennation, when “Hoshioki” (Figure S4B top) and “Shinkawa” workers were used as residents (Figure S4B bottom), respectively, also revealed a significant effect of the treatment (encounter with the “Hoshioki” or “Shinkawa” workers), stemming from no appearance of prolonged-antennation toward *C. japonicus* (Figure S4B top and bottom).

**Figure 6:**
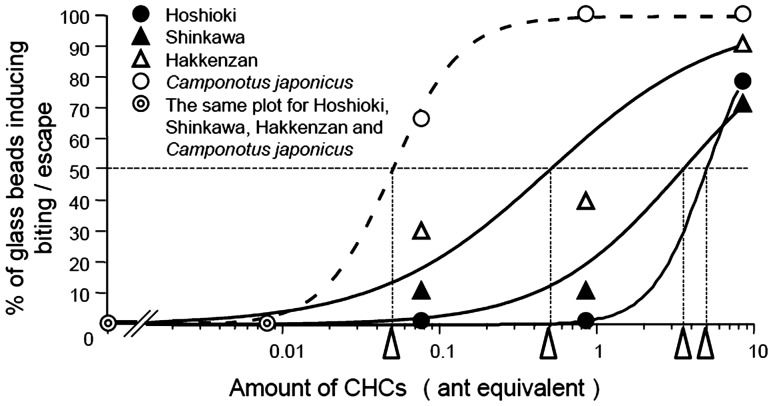
Dose-dependent aggressive response to CHCs. Percentage of discriminator “Hoshioki” workers that bit at the CHC-coating glass beads as surrogate ants are plotted against the ant equivalent amount of CHCs (total amount of components) derived from “Hoshioki”, “Shinkawa”, Hakkenzan”, and *C. japonicus* workers. The fitting curves were drawn by Igor pro ver.4.0, under the hypothesis that all the discriminators could show biting behavior toward infinitely large amounts of CHCs, regardless of their nests. The CHC amounts giving half the maximum response, which is defined as the mean threshold amounts of CHCs for inducing biting behavior, are indicated by arrowheads.

Figure 6 shows fitting curves (Igor pro ver.4.0, Wave Metrics, Inc. OR, USA) describing the biting response of the resident workers to increasing amounts of CHCs that were purified either from Hoshioki”, “Shinkawa”, “Hakkenzan” or *C. japonicus* workers and blindly applied to the glass beads. Considering the mean threshold amount of CHCs as the amount of CHCs that elicits biting behavior in 50% resident workers, it required an estimated 5 ant equivalent of “Hoshioki” CHCs, 3.3 ant equivalent of “Shinkawa” CHCs, 0.5 ant equivalent of “Hakkenzan” CHCs, or 0.05 ant equivalent of *C. japonicus* CHCs. This indicated that the mean threshold amounts of CHCs for eliciting biting behavior of “Hoshioki” workers toward “Shinkawa”, “Hakkenzan” and *C. japonicus* CHCs were 1.5-, 10- and 100-fold lower than that to nestmate CHCs, respectively. Even when we correct for the differences in CHC amounts per ant between *F. yessensis* (17.7±2.1 µg/ant, average±SD, n = 5) and *C. japonicus* (26.5±5.1 µg/ant, average±SD, n = 5), “Hoshioki” workers were still estimated to have 60-fold lower mean threshold amount of CHCs for biting at *C. japonicus* CHCs than at nestmate CHCs.

### Low Genetic Relatedness in the Supercolony

In order to assess the genetic relatedness among the supercolony members, a total of 48 alleles were found at seven microsatellite loci by analyzing 48 ants from each of five nests at “Hoshioki” (34 alleles, heterozygosity Ho = 0.56), “Shinkawa” (27 alleles, Ho = 0.54), “Ishikari” (26 alleles, Ho = 0.61), “Oshoro” (28 alleles, Ho = 0.55), and “Hakkenzan” (28 alleles, Ho = 0.59) (see Figure S6). The genetic analyses revealed that pairwise distances *F*st were as large as 0.06–0.19 (lower diagonal in Table 2) and were statistically significant in all pairs (Table 2 upper diagonal, p<0.001, Fisher’s exact test). The *F*st values were not significantly higher between nests from different colonies than between nests from the same supercolony (p = 0.400, randomization test). This indicates that even within the supercolony, the nests were genetically differentiated from each other.

**Table 2:** Pairwise genetic distance, F_ST_ (lower diagonal), and statistical significance (upper diagonal).

	H	S	I	Ha	O
H		*	*	*	*
S	0.076		*	*	*
I	0.169	0.106		*	*
Ha	0.096	0.160	0.180		*
O	0.063	0.137	0.187	0.125	

H, Hoshioki; S, Shinkawa; I, Ishikari; Ha, Hakkenzan; O, Oshoro.

* Fisher’s exact test, p<0.001.

**Table 3:** Mean value and standard deviation of genetic relatedness within and between nests.

A. Mean relatedness and SD in pairwise comparison within nest					
	H	S	I	Ha	O
mean value	0.107	0.202	0.301	0.205	0.160
SD	0.268	0.270	0.225	0.272	0.258
**B. Mean relatedness (lower diagonal) and SD (upper diagonal)** **in pairwise comparison between nests**					
	**H**	**S**	**I**	**Ha**	**O**
H		0.251	0.254	0.268	0.267
S	0.024		0.267	0.257	0.239
I	−0.097	0.074		0.256	0.251
Ha	−0.019	−0.098	−0.070		0.247
O	0.028	−0.064	−0.100	−0.045	

H, Hoshioki; S, Shinkawa; I, Ishikari; Ha, Hakkenzan; O, Oshoro.

Mean within-nest relatedness±SD within the supercolony ranged from 0.11±0.27 (within Hoshioki nests) to 0.30±0.23 (within Ishikari nests), not different from the within-nest relatedness of nests outside the supercolony. Likewise, there were no apparent differences in mean between nest relatedness when comparing nests within or outside the supercolony, with relatedness varying from −0.10±0.25 (between “Ishikari” and “Oshoro” nests) to 0.07±0.27 (between “Shinkawa” and “Ishikari” nests), respectively (Table 3). Although mean values of the within-nest relatedness were a little higher than between-nest relatedness values, they showed large standard deviations (0.23 to 0.27), indicating that each nest contained many nestmates of low relatedness. Finally, there was no significant correlation either between the Mahalanobis distance and genetic relatedness in Figure S5D (r = −0.18 at p = 0.363) or between frequency of aggressive behavior occurrence toward living ants or CHC-coating glass beads and genetic relatedness (r = −0.24 at p = 0.340 in Figures S5E; r = −0.30 at p = 0.327 in Figure S5F).

## Discussion

### CHC Pattern Discrimination Limited by the CHC Pattern Similarity

Our behavioral data (Figure 5B) showed none or very low aggression between workers within the proposed supercolony, which was not statistically different from that exhibited in encounters between nestmates. Although in one case (encounters between “Hoshioki” and “Tarukawa” workers), aggression between supercolonymates from different nests was higher than that between nestmates, it still tended to be lower than the aggression exhibited by the “Hoshioki” workers toward workers from the two independent nests, “Hakkenzan” and “Oshoro”, which presumably belong to other supercolonies. This corroborates earlier observations [17] that nests of *F. yessensis* are organized in supercolonies. The slightly elevated aggression within the supercolony can be attributed to the observed limited migration distances of workers [26] and consequently limited or no worker exchanges between the two nests. Hence, “Tarukawa” nest can still been considered as a member of the “Ishikari supercolony”. Figure 5B and 5D show experimental results using living ants and surrogates ants made of glass beads, respectively. There is a discrepancy between these two figures on the aggressiveness towards Tarukawa and the Ishikari nests’ workers (Figure 5B) or their CHCs (Figure 5D). Possibly, living ants have other unknown factors than CHCs, which can induce or enhance aggressive behavior.

Nestmate recognition has been shown for several ant species, based on similarity/disparity of CHCs [3], [4], [15], [16], [27], [28]. We therefore, hypothesized that CHC profiles of *F. yessensis* workers are more similar within a supercolony compared to those among nests of independent supercolonies. In order to test the hypothesis we first ascertained that CHCs do indeed constitute the nestmate recognition cues also in *F. yessensis*, verifying this both through chemical comparisons of the CHC profiles and behavioral experiments using the purified CHCs (Figure S5C). Using a well-established behavioral assay, we demonstrated that workers exhibited aggressive behavior toward glass beads coated with purified alien CHCs, which was moreover highly correlated to that exhibited in tests with live ants (Figure S5A). These support the role of CHCs as recognition cues in *F. yessensis*.

Comparison of the CHC profiles revealed that the profile similarity between nests within the supercolony (with an exception of the “Tarukawa” nest) was greater than that between independent nests, each of which represents a different or incipient supercolony. However, even within the supercolony there was a statistical correlation between CHC profile disparity and geographic distance (Figures 2D, 2E and S1B), although the slope for nests within the supercolony was gentler than that between supercolonies. The slight disparity in CHC profiles among members of the supercolony can again be explained by their limited migration range from each resident nest [26]. Thus, the between-nest worker exchange and therefore odor homogenization were limited [29], [30], but at the same time, odor similarity within the supercolony could be maintained as a whole, resulting in the gentle slope of Figure 2D. Nonetheless, such a limited worker exchange might not sufficiently explain for every data on odor similarity.

Although we do not have conclusive evidence of whether environmental or genetic factors are more prominent in shaping the recognition cues, the lack of a clear correlation between the genetic relatedness (Table 3B) and differences in CHC profiles (Figure S5D) among nests indicates that genetic relatedness is not an important determinant in regulating nestmate recognition (see Figures S5E and S5F), but is suggestive of environmental involvement in shaping colony odor. These results are also consistent with previous findings in *Formica exsecta*, in which reduced aggression was linked to decrease rather than increase in diversity of recognition cue [31].

### Putative Role of CHC Sensillum as a Chemosensory Filter

Having demonstrated that similarity/disparity of CHCs is involved in nestmate recognition and tolerance/aggression between supercolonymates, we set to investigate how the sensory system mediates tolerance/aggression switching. The CHC sensillum, which was shown to mediate nestmate recognition in *C. japonicus* [4], was the likely candidate to be examined. In line with the results of chemical and behavioral investigations, the % of sensilla responding to CHCs of non-nestmates within the supercolony was not significantly different from that of nestmates, but the % of sensilla responding to CHCs of non-nestmates outside the supercolony (Oshoro), as well as that of *C. japonicus*, was significantly higher than that of nestmates (Figures 5A). In encounters with *C. japonicus*, 95% CHC sensilla in *F. yessensis* were activated and 95% *C. japonicus* workers were attacked by *F. yessensis* (Figures 5A and 5B). In encounters between supercolonymates, however, such a perfect match between the sensory response and the aggressive behavior expression was not always seen. Despite the low aggression (0–20%) exhibited in these encounters, 28.6–45.6% of the CHC sensilla of “Hoshioki” workers responded to the CHCs of nestmates or non-nestmates from the “Ishikari supercolony”. Assuming that the CHC sensillum filters out nestmate CHC information, this may indicate that the CHC sensillum of *F. yessensis* acts as an “imperfect” chemosensory filter, which allows some of the nestmate or supercolonymate CHC information to pass and be processed in the brain.

An insight on the nature of this chemosensory filter comes from the single sensillar response to reciprocal stimulation with CHCs (Figure 4), in which the impulse patterns displayed either by “Hoshioki” or “Shinkawa” workers indicate activation of a small number of receptor neurons, while the stimulation with *C. japonicus* CHCs elicited impulse generation from a relatively larger number of receptor neurons. We attribute this to desensitization of a fraction of the receptor neurons as a result of internalization of self-CHC blend into the CHC sensilla through the multiple olfactory pores. These receptor neurons become consequently less sensitive to the blend of nestmate or to the very similar blends of supercolonymates, while other receptor neurons for *C. japonicus* specific CHC components preserve their sensitivity. Such receptor desensitization in the CHC sensillum can provide the chemosensory filter acting as a discriminator rather than a neural template. This is further supported by our CHC sensillar recordings with the antennae detached from the head (Figure 4C).

A recent study using EAG and calcium imaging methods in *Camponotus floridanus* [32] showed variable spatial activity patterns in the antennal lobe responding to the antennal stimulation with vapors of not only non-nestmate but also nestmate odors, and suggested that the combination of activated receptor neurons did not provide sufficient information for colony odor discrimination. These results seem to negate our chemosensory filter hypothesis but are not fully consistent with the neural template hypothesis, according to which neural signs induced by repeated stimulations with the nestmate odor should not be so variable as that of non-nestmate odors. Unfortunately, the results of this study cannot be compared with ours because of different methodology adopted. Here we performed single sensillar recording by contact stimulation with CHCs dissolved in the stimulus solution, thus ensuring the exact blend reaches the receptor neurons in the particular type of sensilla putatively responsible for nestmate recognition, rather than broad stimulation of antennae with the vapor emanating from a dummy loaded with CHCs. Resolving these differences awaits further investigation.

The sensory filtration system based on desensitization of receptor neurons can further accommodate the phenomenon observed in several ant species that the CHC profile that provides the colony odor dynamically changes with respect to its relative component ratios [33]–[36]. Probably, internalization of self-CHC blends is instantly achieved, hence the receptor neurons that are responsive to the new self-CHC components are appropriately desensitized. This results in the proper sensory signal passage to the brain without necessitating any changes of neural circuits or their function. In contrast, according to the template hypothesis, recognition of nestmate necessitates concomitant changes of the template in the brain. Nevertheless, this might be able to explain variable neural signs of the nestmate odor observed by Brandstaetter et al. [32].

### Aggressive Behavior Elicitation by Input through the Chemosensory Filter

The importance of the CHC sensillum as a chemosensory filter is in allowing rapid detection of distinct CHC profiles and by that accelerating the subsequent appropriate behavioral response. However unlike in *C. japonicus,* in *F. yessensis*, some nestmate information passed through “imperfect” chemosensory filter but did not induce aggressive behavior (Figure 5). Hence, we propose the existence of a brain center responsible for switching on aggressive behaviors. Although this kind of center could be considered to hold the “template” for the colony odor, here we simply hypothesize that the putative center sets a threshold and is activated by above-threshold neural inputs coming through the chemosensory filter. Comparing the sensillar response with the aggressive behavior expression in Figure 5, the range of such a threshold for activation of the behavior-switching center can be estimated. Stimulation with nestmate’s odor with no induction of aggressive behaviors elicited 28% of the sensilla responding, which was evidently below the threshold. When stimulated with the “Tarukawa” CHC blend, for example, 45% of the sensilla responded and about 20% of the tested encounters revealed aggressive behavior. This suggested that the neural input from active 45% sensilla was above the threshold in the 20% individuals but not in others. Thus, the behavioral response is subjected to individual variation among workers. In the encounters with workers of independent supercolonies, merely 30–40% of the encounters were aggressive, but all of these 30–40% encounters culminated in the death of invaders. It was then presumed that once stimulated at above-threshold level, the behavior-switching center triggered aggressive behavior in an all-or none manner.

The neural response from the CHC sensilla further suggests that the hypothetical behavior-switching center is tuned not only to the number of activated sensilla but also to characteristic of their responsiveness (Figures 3, 4 and S3). The CHC sensilla, when stimulated with the CHCs of nestmates or supercolonymates, showed a weak response with sparse impulses, in contrast to a stronger response with vigorous impulses to CHCs of non-supercolonymates or allospecific ants (*C. japonicus*). This differential response was not due to the existence of two functionally different types of CHC sensilla, because the same sensillum occasionally responded with different appearances of impulse arrays, depending on the stimuli. Considering the “one receptor gene for one receptor neuron rule” in olfactory receptor neurons [37], it is possible that these differences are due, rather, to the number of receptor neurons expressing specific genes and generating specific impulse units, respectively in a recording sensillum (see Figures 4 and S3). Our electrophysiological data by the single sensillar recordings suggest that the effect of CHCs on impulse unit recruitment is higher with allospecific CHCs than with conspecific CHCs, but whether it is higher in those from nests outside the supercolony compared to conspecific CHCs from nests inside the supercolony is still equivocal.

Such a behavior-switching system can allow experience- or context-dependent modulation of the behavioral expression toward alien CHCs possibly by resetting the threshold via hormonal and/or neural regulations [38], [39]. The previous reports in *Pristomyrmex pungens* [40] and *Cataglyphis fortis* [41] showed that workers exhibit greater aggressive behavior toward familiar non-nestmates than toward unfamiliar non-nestmates. This phenomenon cannot fully be explained by the traditional template for nestmate recognition. It is presumed that memories formed by repetitive experiences of aggressive behavior expression toward competitors on foods, etc. influenced their recognition system. The external cues required for forming and/or recalling such memories are elusive, but every modality of information about competitors or every context to learn from competition seems possible.

A recent report in the carpenter ant *Camponotus floridanus* [42] provides evidence supporting the neural template hypothesis rather than the chemosensory filter hypothesis. They have shown that masking the antennae with an alien odor did not change nestmate recognition in the short term but had a long-term effect and claimed that it takes proper time for reformation process of the neural template. However, they did not present any data on how the treatment affected the antennal chemosensory neurons. Furthermore, both their bioassay and scoring of aggression was different from ours, making it hard to compare their experimental data with ours.

On the other hand, Figure 6 indicates that excess amounts of CHCs, regardless of nestmate or non-nestmate CHCs, induce aggressive behavior in workers. According to our chemosensory filter hypothesis based on desensitization of receptor neurons, excess amount of nestmate CHCs can overcome the desensitization filter to activate the receptor neurons for nestmate CHC components and the corresponding glomeruli, resulting in aggressive behavior expression. Thus, the induced aggressive behavior is hardly explained by the template hypothesis.

In summary, we have demonstrated here that the unicolonial species, *F. yessensis*, exhibits very low frequency of aggressive behavior occurrence among workers within the supercolony. We have further demonstrated, using purified CHCs, that the CHCs serve as recognition cues for eliciting aggressive behavior and congruent among workers of the same supercolony. Our data of the single sensillar recordings in the CHC sensilla indicated that subtle differences in CHC profile among nests within a supercolony were hardly discriminated by the CHC sensilla, whereas CHCs originated from workers outside the supercolony elicited distinct sensillar response and so did allospecific CHCs. In conclusion, we consider the best hypothesis to explain our results as follows; In respect of similarity/disparity of the CHC profile, discrimination of nestmates or supercolonymates from others is attained primarily at the antennal chemosensilla as peripheral filters and subsequently at a brain center responsible for triggering aggressive behavior, which can be activated only by the above-threshold inputs. This might underlie the maintenance of unicolonial society with little expression of conspecific aggression in a supercolony-forming ant, *F. yessensis*.

## Materials and Methods

### Ants

For our study, after behavioral tests in the field to assess whether the nests belonged to the supercolony or lay outside it [17], we selected four different nest sites within the “Ishikari supercolony”: “Hoshioki”, “Shinkawa”, “Tarukawa”, and “Ishikari”, all within a perimeter of 10 km; and two additional nest sites, “Hakkenzan” and “Oshoro”, which contained polygyne colonies that did not belong to the “Ishikari supercolony”. “Oshoro” comprised part of a smaller supercolony compared to the “Ishikari supercolony”, while “Hakkenzan” seemed to be a newly-founded nest outside the supercolony. Colony fragments (containing workers, queens, and brood) used for chemical analyses, electrophysiological experiments or behavioral experiments in the laboratory, which were collected after the field behavioral experiments from four different nests within the “Ishikari supercolony” and two other independent colonies from sites outside the “Ishikari supercolony”. These two colonies are different from the “Ishikari supercolony” in size but are still highly polygynous. They were kept in the laboratory in separate plastic boxes (35 × 25.5 × 3 cm), respectively, and fed on 30% honey and water.

### CHC Analysis

For the CHC analysis, individual ants were immersed in 1 ml *n*-hexane for 5 min for extraction, and the extract was further purified on a silica gel (230–400 mesh, Merck Ltd., Tokyo, Japan) column (diameter, 0.5 cm; length, 8.5 cm) to obtain a pure CHC fraction. The purified CHCs were recovered in 5 ml *n*-hexane, concentrated to several microliters, and stored at −20°C until gas chromatography (GC) analysis. Before analysis, the CHC samples were dissolved in 30 µl *n*-hexane, and 1 µl was injected to a Shimadzu GC-14B GC equipped with a flame ionization detector. Compound separation was achieved using a DB-1HT non-polar capillary column (diameter, 0.25 mm; length, 15 m; film thickness, 0.1 µm) that was temperature programmed from 60°C (1 min hold) to 210°C at 30°C/min, and from 210 to 350°C at 10°C/min with a final hold of 5 min. Helium was used as the carrier gas, at a column head pressure of 100 kPa. Injection was made directly onto the capillary column using an on-column injector that was temperature programmed from 60°C (0.01 min hold) to 350°C at 15°C/min, and then held at the final temperature for 24 min. The amounts of single CHCs were calculated by peak integration.

GC-MS analysis was carried out with a JMS-700 using EI mode at 70 eV, and equipped with a polar capillary column (VF-1 ms, VARIAN, length, 15 m; diameter, 0.32 mm; film thickness, 0.1 µm). The column oven was operated under the conditions described above, but the injection mode was splitless. Compound identifications were deduced from their mass fragmentation patterns.

### Discriminant Analysis

The discriminant analysis was conducted with 10 workers each from the “Hoshioki”, “Shinkawa”, “Tarukawa”, “Hakkenzan”, and “Oshoro” nests, respectively, and 9 workers from the “Ishikari” nest. We used the 25 CHC peaks indicated by A–Y in Figure 2A (see Table1), which were commonly observed in all individual samples of 59 workers from these 6 nests, the relative area of which was standardized to all peak areas (100%), and transformed following Aitchinson’s formula [43]. We present the results of discriminant analyses in two separate panels as shown in Figures 2B and 2C. For these figures, the transformed values were first subjected to a principal component analysis, of which the two discriminant functions were extracted (discriminant function 1 explaining 68.41 (Figure 2B) and 85.27% (Figure 2C) and discriminant function 2 explaining 20.06 (Figure 2B) and 14.73% (Figure 2C) at p = 0.02 (Figure 2B) and <0.01 (Figure 2C), respectively), were used as variables in the discriminant analysis. Thus, the Eigenvalues were 8.26 (Figure 2B) and 38.18 (Figure 2C), and the Wilks’ lambdas were 0.013 (Figure 2B) and 0.003 (Figure 2C), respectively. Alternatively, if we attempted a discriminant analysis in a single panel with all the data, the variances of discriminant functions 1 and 2 decreased to 40.84 and 24.40%, respectively (p>0.05). Then, the Eigenvalue decreased to 2.2 and the Wilks’ lambda increased to 0.033. Therefore, compared to such a discriminant analysis in a single panel, the discriminant analysis in two separate panels as shown in Figures 2B and 2C provide greater statistical accuracy with lower p value (P<0.05), higher Eigenvalue and lower Wilks’ lambda.

Mahalanobis distances from a group centroid to the position in the plot of each individual of the tested nests were calculated by software, JMP ver. 7, and statistically compared by Tukey HSD test, using SPSS ver. 15.

### Scanning Electron Microscopy

Whole bodies of *F. yessensis* were dehydrated in acetone for >2 days. After a brief sonication for cleaning, the whole body and the CHC sensilla on the antennae were observed under a scanning electron microscope (SU-1500, Hitachi, Japan). For counting the sensilla, newly-emerged workers were used. The isolated antennae were dehydrated with an ethanol series (50, 70, 80, 90, 95 and 100%), cleaned by sonication and stored in acetone until SEM observation.

### Electrophysiological Recording

The isolated head of a “Hoshioki” worker was connected with a platinum indifferent electrode under a microscope. The electrophysiological response was recorded from the CHC sensillum by the tip-recording method, in which the target CHC sensillum was capped with the recording electrode of a glass capillary filled with stimulus solution, in accordance with Ozaki’s method [4]. The CHCs used for the stimulus solutions were extracted with *n*-hexane and purified on a silica gel column as mentioned above for the CHC analysis. Before electrophysiological experiments on the same day, the CHC stimulus solution was prepared by dissolving the CHCs in 10 mM NaCl with 0.1% Triton X-100, to a final concentration of 0.8–1 ant equivalent CHCs/100 µl. The stimulus duration was 10 s. Each sensillum was used only once, and at least 20 sensilla of at least four “Hoshioki” workers, randomly chosen from our breeding box, were tested for each CHC stimulus. We recorded the response of the same sensillum, which received pairwise stimulation; the sensillum was first stimulated with 10 mM NaCl with 0.1% Triton X-100, and after >3 min interval, stimulated with one of the test CHCs dissolved in 10 mM NaCl with 0.1% Triton X-100. In the experiments using the CHC sensilla of “Shinkawa” and “Hoshioki”, stimulus presentation was done blindly. For recording from the CHC sensilla on the antenna detached from the head, the “Hoshioki” antenna was cut at the middle of the scape segment and connected with an indifferent glass capillary electrode filled with ant linger solution (130 mM NaCl, 6 mM KCl, 4 mM MgCl_2_, 160 mM Sucrose, 10 mM HEPES, 5 mM CaCl_2_). Such isolated antennae, which are free from feedback control by the brain, can be alive and vigorously respond to CHC blend of *C. japonicus* as a positive control stimulus at least for 1 h.

The recorded impulses of various shapes were sorted into unit clusters through a 100–1800 Hz band-pass filter, using SpikeTaro (Chinou Jouhou Shisutemu Inc., Kyoto, Japan. http://www.chino-js.com/ja/service-engineer.html) [44] modified so that even partially overlapping impulses were recognized as separate and sorted accordingly as different units. Spike Taro is an originally custom-developed, but now commercially obtainable impulse sorting software for multi-units neural impulses, which is programmed based on C language (C++Builder ver. 5.0, Borland Corporation, TX, USA) with the sorting algorithm on the basis of two criteria; impulse amplitude and wave form correlation coefficient. In this program, the impulses having amplitude within ±10% wave-form coefficient over 0.98% were considered to be similar and to be generated from the same neuron. Recording waves with an appearance probability of less than 1% were not counted as reliable impulse units in our Raster plot (Figure S3).

### Behavioral Experiments

These were carried out in the field at the “Hoshioki” site in July and August 2006, June 2007 and, June, 2009. On the day of each assay, 30 workers each were collected from the “Shinkawa”, “Tarukawa”, “Ishikari”, “Hakkenzan”, and “Oshoro” nests and marked with a silver paint marker on the dorsal surfaces of their abdominal cuticle. They were placed in a box containing small amounts of nest material and transported by car to the “Hoshioki” nest site, to serve as invaders. Tests of the supercolonymates as invaders at the “Hoshioki” nest were conducted on the same day of collection. However, since it was impossible to transport the alien ants from two remote localities on the same day, we tested the alien ants from one locality at a time, consequently not blindly. At the onset of each test we gently placed a randomly chosen invader worker at a point <30 cm away from a particular “Hoshioki” nest entrance. The same “Hoshioki” nest was used for all the field behavioral tests. One invader worker was presented at a time, and observations lasted until it had made contact with five different “Hoshioki” workers. Thus, the interval between invader presentations was not constant. We recorded the number of invader individuals that were either bitten or antennated for a prolonged time by at least one of the five “Hoshioki” workers. Once the “Hoshioki” worker had interacted with the invader it was immediately removed in order to avoid double counting of reacting “Hoshioki” workers during the series of experiments, in which 30 invaders from each locality were tested. Introductions of 30 “Hoshioki” workers into their own nest site served as control. We also introduced 21 *C. japonicus* workers in order to assess the interspecific aggression.

The field behavioral experiments were complemented with blind laboratory assays. In these assays we used “Hoshioki” as well as “Shinkawa” workers as discriminators, each of which encountered 10 individual *F. yessensis* invaders, derived from “Hoshioki”, “Shinkawa”, and “Hakkenzan” nests, and 10 individual *C. japonicus* workers.

These experiments were conducted during day time between 10∶00 and 15∶00. At least one hour before the experiments, we set an observation arena surrounded by a circular plastic wall (diameter, 6 cm; height, 3 cm), both sides of which was thinly covered with talc powder to prevent ants from escaping and intruding. A discriminator ant was put in the arena and left until it calmed down (at least for 5 min), and then an invader ant was gently transferred into the arena by using a tooth-pick. Upon the first interaction, we recorded both biting and prolonged-antennation exhibited by the discriminator toward the invader. We repeated that test for each invader with 5 different discriminators, which were subsequently encountered with an invader with 5 min intervals, thus obtaining the behavioral data of 5 discriminators toward the same invader. All the experiments were conducted double blindly; the observer-scorer who had no information about invader ants and the introducer who had no information about discriminator ants and randomly selected the invader ant for each assay.

The role of purified CHC in nestmate recognition was tested in the laboratory using CHC-coating glass beads (4 mm in diameter), according to Ozaki [4]. The glass beads were coated with 0.8 ant equivalent of CHCs, previously extracted and purified through a silica gel column as mentioned above for the CHC analysis, dissolved in 10 µl *n*-hexane, and then exposed for at least 10 min, to ensure full solvent evaporation, before being presented to the “Hoshioki” workers. For the blind tests, the prepared glass beads were marked and shuffled. On the following day, we conducted the blind experiments using the same 30 “Hoshioki” workers as discriminators, presenting one of the glass beads to each discriminator worker 5 times with 5 min intervals. When the discriminator workers bit the glass beads at least once, a positive score was given.

For the dose-dependent behavioral response test using CHC-coating glass beads, we employed the same behavioral assay with CHC amount of 8, 0.8, 0.08 or 0.008 ant equivalents.

### Analysis of Genetic Structure

DNA was extracted from 48 ants collected at each nest of “Hoshioki”, “Shinkawa”, “Ishikari”, “Hakkenzan”, and “Oshoro” using PUREGENE DNA isolation Kit (Qiagen Japan, Tokyo, Japan) and amplified at seven microsatellite loci, which have been developed for *F. yessensis* (Fy3, Fy4, Fy13, Fy15, [45]), *Formica. lugubris* (FL12, FL21, [46]), and *Formica. exsecta* (FE17, [47]). The amplification by multiplex PCR was performed in 10 µl solution containing 1.0 µl of PCR buffer, 0.2 mM of dNTP, 0.1–0.4 µM of each primer (forward primer labeled with fluorescent FAM, NDE or PET), 0.15 units of Taq DNA polymerase (Sigma-Ardrich Japan K. K. Tokyo, Japan), 0.2–0.3 µl of template DNA and 6.87–8.07 µl of dH_2_O. PCR condition was 94°C for 3 min followed by 28–35 cycles of 94°C for 30 sec or 1 min, 44–52°C for 30 sec or 1 min, 72°C for 30 sec or 1 min, and final extension at 72°C for 10 min. The size of each amplified region was determined by ABI Prism 3100 Genetic Analyzer and the program GEVESCAN 3.7 (Applied Biosystems Inc. CA, USA). The sample size (n = 48 workers) at each nest seemed sufficient for the analysis of genetic structure because the total number of alleles at the seven loci reached a plateau at n = 30–35 workers (see Figure S4). Based on the number of alleles, gene diversity was represented by observed heterozygosity, Ho, which was not significantly different from the heterozygosity expected from Hardy-Weinberg equilibrium in each nest. The genetic distance between pairwise nests was examined by *F*st [48], using FSTAT 2.9.3.2 [49] and Fisher’s exact test using GENEPOP [50]. Between- and within-nest relatedness were calculated after Queller and Goodnight (1989) [51] using the program KINGOUP v2_01212b.

## Supporting Information

Figure S1 **Discriminant analyses of the CHC profiles (A) and relationships between Mahalanobis distance and geographic distance (B).** (**A**) Discriminant analyses using the 2 independent nests and either “Shinkawa”, “Tarukawa” or “Ishikari” from the “Ishikari supercolony”, employing the same parameters as in Figure 2C. (**B**) Correlation between geographic distance and the Mahalanobis distance (average±SE) between the 2 independent nests and either “Shinkawa”, “Tarukawa” or “Ishikari” from the “Ishikari supercolony”.
(PPT)

Figure S2 **Comparison of the Mahalanobis distances, based on the discriminant analyses of the CHC profiles.** (**A**) Among the 4 nests within the “Ishikari supercolony”; (**B**) Among the 3 independent nests: “Hoshioki” within the “Ishikari supercolony”, and “Oshoro” and “Hakkenzan” outside the “Ishikari supercolony”. Each column indicates the average Mahalanobis distance from the nest centroid (indicated by underlined letter) to individual data plots of nests within (white columns) and outside the “Ishikari supercolony” (hatched columns). Single and double asterisks indicate significant difference from the within-nest variation in the Mahalanobis distance of the underlined nests at P<0.05 and <0.001, respectively (F = 11.94 for the graph underlining “Hoshioki”, F = 36.37 for the graph underlining “Shinkawa”, F = 14.52 for the graph underlining “Tarukawa”, F = 33.23 for the graph underlining “Ishikari” in (A); F = 434.39 for the graph underlining “Hoshioki”, F = 175.61 for the graph underlining “Hakkenzan”, F = 390.86 for the graph underlining “Oshoro” in the top panels of (B); F = 288.07 for the graph underlining “Shinkawa”, F = 402.73 for the graph underlining “Hakkenzan”, F = 203.29 for the graph underlining “Oshoro” in the second panels from the top of (B); F = 2070.93 for the graph underlining “Tarukawa”, F = 9805.86 for the graph underlining “Hakkenzan”, F = 6457.37 for the graph underlining “Oshoro” in the third panels from the top of (B); F = 3756.91 for the graph underlining “Ishikari”, F = 5213.13 for the graph underlining “Hakkenzan”, F = 3826.51 for the graph underlining “Oshoro” in the bottom panels of (B); Tukey HSD test). The degrees of freedom are 38 for (**A**), 28 for the 3 bottom panels of (B), and 29 for other panels in (B). Vertical bars represent the standard errors.
(PPT)

Figure S3 **Raster plots for the experiments presented in** **Figure 4A and 4B** **.** Impulses of the “Hoshioki” CHC sensilla on the antennae attached to the haeds (**A**) and “Shinkawa” CHC sensilla on the antennae attached to the haeds (**B**) and those of the “Hoshioki” CHC sensilla on the antennae detached from the haeds (**C**), which are sorted into different unit clusters, are plotted against time after beginning of stimulation. H-S-1 and 2, e.g., indicate clusters 1 and 2 corresponding to different shapes of impulse units that were sorted from the impulse train recorded in a “Hoshioki” sensillum stimulated with “Shinkawa” CHCs; H-s-1 to 4, e.g., indicate clusters 1 to 4 corresponding to different shapes of impulse units sorted from the impulse train recorded in the same “Hoshioki” sensillum stimulated with solvent without “Shinkawa” CHCs. Left, Raster plots for the representative electrophysiological responses to CHCs extracted from “Hoshioki”, “Shinkawa”, “Hakkenzan”, and *C. japonicus*. Right, Raster plots for control recordings of the same sensilla in response to the solvent, 10 mM NaCl plus 0.1% Triton X-100.
(PPT)

Figure S4 **Blind experiments on aggressive behavior.** (**A**) Percentage of workers from “Hoshioki” (H), “Shinkawa” (S), “Hakkenzan” (Ha) *F. yessensis* and *C. japonicus* (*C*) (out of total workers tested, respectively) that induced biting behavior of “Hoshioki” (upper plate) and “Shinkawa” workers (lower plate). (**B**) Percentage of workers from the same nests of *F. yessensis* and *C. japonicus* that induced prolonged-antennation behavior of “Hoshioki” (upper plate) and “Shinkawa” workers (lower plate). Resident nests are marked by under bars. Different letters indicate significant differences by Tukey WSD test.
(PPT)

Figure S5 **Correlates of biting behaviors, Mahalanobis distances of CHC profiles, and genetic relatedness in resident or invader ants.** (**A**) Correlation between % of invader ants and of glass beads inducing biting behavior of resident “Hoshioki” ants. (**B**) Correlation between aggressive behavior and average of Mahalanobis distances of CHC profiles between resident “Hoshioki” ants and invader ants. (**C**) Same as (**B**) but % of glass beads inducing biting instead of % of ants inducing biting. (**D**) Correlation between chemical similarity (Mahalanobis distances of CHC profiles) and genetic relatedness between resident and invader ants. (**E**) Correlation between genetic relatedness and aggressive behavior in encounters involving resident “Hoshioki” and invader ants. (**F**) Same as (**E**) but % of glass beads inducing biting instead of % of ants inducing biting. Indicated r-values are correlation coefficients. The straight lines are regression lines drawn by the least-squares method.
(PPT)

Figure S6 **Coleman rarefaction curve for “Hoshioki” workers.** For the analysis of genetic structure, 48 ant individuals were used. The total number of alleles at seven microsatellite loci was counted by randomly choosing each number of individuals (n) from the 48 ants, and the mean number of alleles with standard deviation (bar) was calculated from 1,000 replications. The curve reaches plateau around n = 30, indicating that the sample size 48 is large enough for the analysis of genetic structure. In all five nests, the Coleman rarefaction curve reached plateau at n = 30–35 (data not shown).
(PPT)

Table S1 **Average number of sensilla basiconica (CHC sensilla) in a worker’s antenna.**
(PPT)

## References

[1] Price WP (1975) Insect ecology: New York: Wiley. 514 p.

[2] Hölldobler B, Wilson OE (1990) The ants. Cambridge: Belknap Press of Harvard University Press. 732 p.

[3] Akino T, Yamamura K, Wakamura S, Yamaoka R (2004) Direct behavioral evidence for hydrocarbons as nestmate recognition cues in *Formica japonica* (Hymenoptera: Formicidae). Appl Entomol Zool 39: 381–387.

[4] Ozaki M, Wada-Katsumata A, Fujikawa K, Iwasaki M, Yokohari F, et al (2005) Ant nestmate and non-nestmate discrimination by a chemosensory sensillum. Science 309: 311–314. 15947139 10.1126/science.1105244

[5] Martin SJ, Vitikainen E, Helanterä H, Drijfhout FP (2008) Chemical basis of nest-mate discrimination in the ant *Formica exsecta* . Proc R Soc B 275: 1271–1278. 10.1098/rspb.2007.1708 PMC2602672 18319215

[6] Holway AD, Lach L, Suarez AV, Tsutsui ND, Case TJ (2002) The causes and consequences of ant invasions. Ann Rev Ecol Syst 33: 181–233.

[7] Holway AD, Suarez VA, Case JT (1998) Loss of intraspecific aggression in the success of a widespread invasive social insect. Science 282: 949–952. 9794767 10.1126/science.282.5390.949

[8] Tsutsui DN, Suarez VA, Holway AD, Case JT (2000) Reduced genetic variation and the success of an invasive species. Proc Natl Acad Sci USA 97: 5948–5953. 10811892 10.1073/pnas.100110397 PMC18539

[9] Giraud T, Pedersen SJ, Keller L (2002) Evolution of supercolonies: The Argentine ants of southern Europe. Proc Natl Acad Sci USA 99: 6075–6079. 11959924 10.1073/pnas.092694199 PMC122904

[10] Pedersen SJ, Krieger JBM, Vogel V, Giraud TV, Keller L (2006) Native supercolonies of unrelated individuals in the invasive Argentine ant. Evolution 60: 782–791. 16739459

[11] Wetterer JK, Walsh PD, White LJT (1999) *Wasmannia auropunctata* (Roger) (Hymenoptera: Formicidae), a destructive tramp-ant, in wildlife refuges of Gabon. African Entomol 7: 292–294.

[12] Jourdan H, Sadlier RA, Bauer AM (2001) Little fire ant invasion (*Wasmannia auropunctata*) as a threat to New Caledonian lizards: Evidences from a sclerophyll forest (Hymenoptera: Formicidae). Sociobiology 38: 283–301.

[13] Vonshak M, Dayan T, Foucaud J, Estoup A, Hefetz A (2009) The interplay between genetic and environmental effects on colony insularity in the clonal invasive little fire ant *Wasmannia auropunctata* . Behav Ecol Sociobiol 63: 1667–1677.

[14] Vonshak M, Dayan T, Ionescu-Hirsh A, Freidberg A, Hefetz A (2010) The little fire ant *Wasmannia auropunctata*: a new invasive species in the Middle East and its impact on the local arthropod fauna. Biol Invasions 10.1007/s10530–009–9593–2.

[15] Silverman J, Liang D (2001) Colony disassociation following diet partitioning in a unicolonial ant. Naturwissenshaften 88: 73–77. 10.1007/s001140000198 11320891

[16] Torres WC, Brandt M, Tsutsui DN (2007) The role of cuticular hydrocarbons as chemical cues for nestmate recognition in the invasive Argentine ant (Linepithema humile). Insect Soc 54: 363–373.

[17] Higashi S (1976) Nest proliferation by budding and nest growth pattern in *Formica* (*Formica*) *yessensis* in Ishikari shore. Jour Fac Sci Hokkaido Univ Ser VI Zool 20: 359–389.

[18] Higashi S, Yamauchi K (1979) Influence of a supercolonial ant *Formica* (*Formica*) *yessensis* Forel on the distribution of other ants in Ishikari coast. Jap J Ecol 29: 257–264.

[19] Cherix D (1980) Note preliminaire sur la structure, la phenologie et le regime alimentaire d’une super-colonie de *Formica lugubris Zett* . Insectes Soc 27: 226–236.

[20] Holzer B, Chapuisat M, Kremer N, Finet C, Keller L (2006) Unicoloniality, recognition and genetic differentiation in a native *Formica* ant. Euro Soc Evol Biol 19: 2031–2039. 10.1111/j.1420-9101.2006.01133.x 17040400

[21] Buczkowski G (2010) Extreme life history plasticity and the evolution of invasive characteristics in a native ant. Biolo Invasions 12: 3343–3349.

[22] Leniaud L, Hefetz A, Grumiau L, Aron S (2011) Multiple mating and supercoloniality in *Cataglyphis* desert ants. Biol J Linean Soc 104: 866–876.

[23] Bonavita-Cougourdan A, Clément LJ, Lange C (1986) Nestmate recognition: the role of cuticular hydrocarbons in the ant *Camponotus vagus scop* . J Entomol Soc 22: 1–10.

[24] De Maesschalck R, Jouan-Rimbaud D, Massart LD (2000) The Mahalanobis distance. Chemometr Intell Lab 50: 1–18.

[25] Ugelvig VL, Drijfhout PF, Kronauer JCD, Boomsma JJ, Pedersen SJ, et al. (2008) The introduction history of invasive garden ants in Europe: Integrating genetic, chemical and behavioral approaches. BMC Biol 10.1186/1741-7007-6-11 10.1186/1741-7007-6-11 PMC2292682 18302731

[26] Higashi S (1978) Task and areal conservatism and internest drifting in a red wood ant Formica (Formica) yessensis Forel. Jpn J Ecol 28: 307–317.

[27] Brandt M, van Wilgenburg E, Sulc R, Shea JK, Tsutsui DN (2009) The scent of supercolonies: the discovery, synthesis and behavioral verification of ant colony recognition cues. BMC Biol 10.1186/1741-7007/7/71 10.1186/1741-7007-7-71 PMC2775022 19863781

[28] Guerrieri FJ, Nehring V, Jørgensen CG, Nielsen J, Galizia CG, et al (2009) Ants recognize foes and not friends. Proc Biol Sci 276: 2461–2468. 19364750 10.1098/rspb.2008.1860 PMC2690455

[29] Dahbi A, Cerda X, Hefetz A, Lenoir A (1997) Adult transport in the ant *Cataglyphis iberica*: a means to maintain a uniform colonial odour in a species with multiple nests. Physiol Entomol 22: 13–19.

[30] Dahbi A, Lenoir A (1998) Nest separation and the dynamics of the Gestalt odor in the polydomous ant *Cataglyphis iberica* (Hymenoptera, Formicidae). Behav Ecol Sociobiol 42: 349–355.

[31] Martin SJ, Helanterä H, Kiss K, Lee YR, Drijfhout FP (2009) Polygyny reduces rather than increases nestmate discrimination cue diversity in *Formica exsecta* ants. Insect Soc 56: 375–383.

[32] Brandstaetter AS, Rösser W, Kleinaidam CJ (2011) Friends and foes from an ant brain’s point of view-Neronal correlations of colony odors in a social insect. PloS ONE 6: e21383. 21731724 10.1371/journal.pone.0021383 PMC3121771

[33] Vander Meer RK, Saliwanchik D, Lavine B (1989) Temporal changes in colony cuticular hydrocarbon patterns of *Solenopsis invicta* . J Chem Ecol 15: 2115–2125. 24272300 10.1007/BF01207442

[34] Provost E, Riviere G, Roux M, Morgan DE, Bagneres GA (1993) Change in the chemical signature of the ant *Leptothorax lichtensteini* bondroit with time. Insect Biochem Mol Biol 23: 945–957.

[35] Boulay R, Hefetz A, Soroker V, Lenoir A (2000) *Camponotus fellah* colony integration: worker individuality necessitates frequent hydrocarbon exchanges. Anim Behav 59: 1127–1133. 10877891 10.1006/anbe.2000.1408

[36] Lahav S, Soroker V, Vander Meer RK, Hefetz A (2001) Segregation of Colony Odor in the Desert Ant *Cataglyphis niger* . J Chem Ecol 27: 927–943. 11471945 10.1023/a:1010382919227

[37] Hildebrand GJ, Shepherd MG (1997) Mechanisms of olfactory discrimination: Converging evidence for common principles across phyla. Ann Rev Neurosci 20: 595–631. 9056726 10.1146/annurev.neuro.20.1.595

[38] Boulay R, Katzav-Gozansky T, Vander Meer RK, Hefets A (2003) Colony insularity through queen social motivation in ants. Proc R Soc Lond, Ser B: Biol Sci 270: 971–977. 10.1098/rspb.2002.2325 PMC1691331 12803913

[39] Roeder T (2005) Tyramine and octopamine: Ruling behavior and metabolism. Ann Rev Entomol 50: 447–477. 15355245 10.1146/annurev.ento.50.071803.130404

[40] Knaden M, Wehner R (2003) Nest Defense and Conspecific Enemy Recognition in the Desert Ant *Cataglyphis fortis* . J Insect Behav 16: 717–730.

[41] Sanada-Morimura S, Minai M, Yokoyama M, Hirota T, Satoh T, Obara Y (2003) Encounter-induced hostility to neighbors in the ant *Pristomyrmex pungens* . Behav Ecol 14: 713–718.

[42] Leonhardt SD, Brandstaetter AS, Kleineidam CJ (2007) Reformation process of the neural template for nestmate-recognition cues in the carpenter ant *Camponotus floridanus* . J Comp Physiol A 193: 993–1000. 10.1007/s00359-007-0252-8 17639411

[43] Aitchinson J (1986) The statistical analysis of compositional data: Blackburn Press.

[44] Goodwyn PP, Wada-Katsumata A, Okada K (2009) Morphology and neurophysiology of tarsal vibration receptors in the water strider *Aquarius paludum* (Heteroptera: Gerridae). J Insect Physiol 55: 855–861. 19523956 10.1016/j.jinsphys.2009.06.001

[45] Hasegawa E, Imai S (2004) Characterization of microsatellite loci in red wood ants *Formica* (s. str) spp. and the related genus Polyergus. Mol Ecol Notes 4: 200–203.

[46] Chapuisat M (1996) Characterization of microsatellite loci in *Formica lugubris* B and their variability in other at species. Mol Ecol 5: 599–601. 8794567 10.1111/j.1365-294x.1996.tb00354.x

[47] Gyllenstrand N, Gertsch PJ, Pamilo P (2002) Polymorphic microsatellite DNA markers in the ant *Formica excecta* . Mol Ecol Notes 2: 67–69.

[48] Weir BS, Cockerham CC (1984) Estimating F-statistics for the analysis of population structure. Evolution 38: 1358–1370. 28563791 10.1111/j.1558-5646.1984.tb05657.x

[49] Goudet (2001) FASTAT, a program to estimate and test gene diversities and fixation indices (version 2.9.3). 42. Raymond F, Rousset M (1995) Testing heterozygote excess and deficiency. Genetics 140: 1413–1419. 10.1093/genetics/140.4.1413 PMC1206704 7498780

[50] Queller DC, Goodnight KF (1989) Estimating relatedness using genetic markers. Evolution. 43: 258–275. 28568555 10.1111/j.1558-5646.1989.tb04226.x

[51] Konovalov DA, Manning C, Henshaw MT (2004) KINGROUP: a program for pedigree relationship reconstruction and kin group assignments using genetic markers. Mol Ecol Notes 4: 779–782.

