# Supplementary material for: Chemical Discrimination and Aggressiveness via Cuticular Hydrocarbons in a Supercolony-Forming Ant, Formica yessensis
Source: PLoS One. 2012 Oct 24;7(10):e46840. doi: 10.1371/journal.pone.0046840 (PMC3480379; doi:10.1371/journal.pone.0046840)
Supplement: Figure S1 — Discriminant analyses of the CHC profiles (A) and relationships between Mahalanobis distance and geographic distance (B). (A) Discriminant analyses using the 2 independent nests and either “Shinkawa”, “Tarukawa” or “Ishikari” from the “Ishikari supercolony”, employing the same parameters as in Figure 2C. (B) Correlation between geographic distance and the Mahalanobis distance (average±SE) between the 2 independent nests and either “Shinkawa”, “Tarukawa” or “Ishikari” from the “Ishikari supercolony”. (PPT) [file pone.0046840.s001.ppt]

## Slide 1
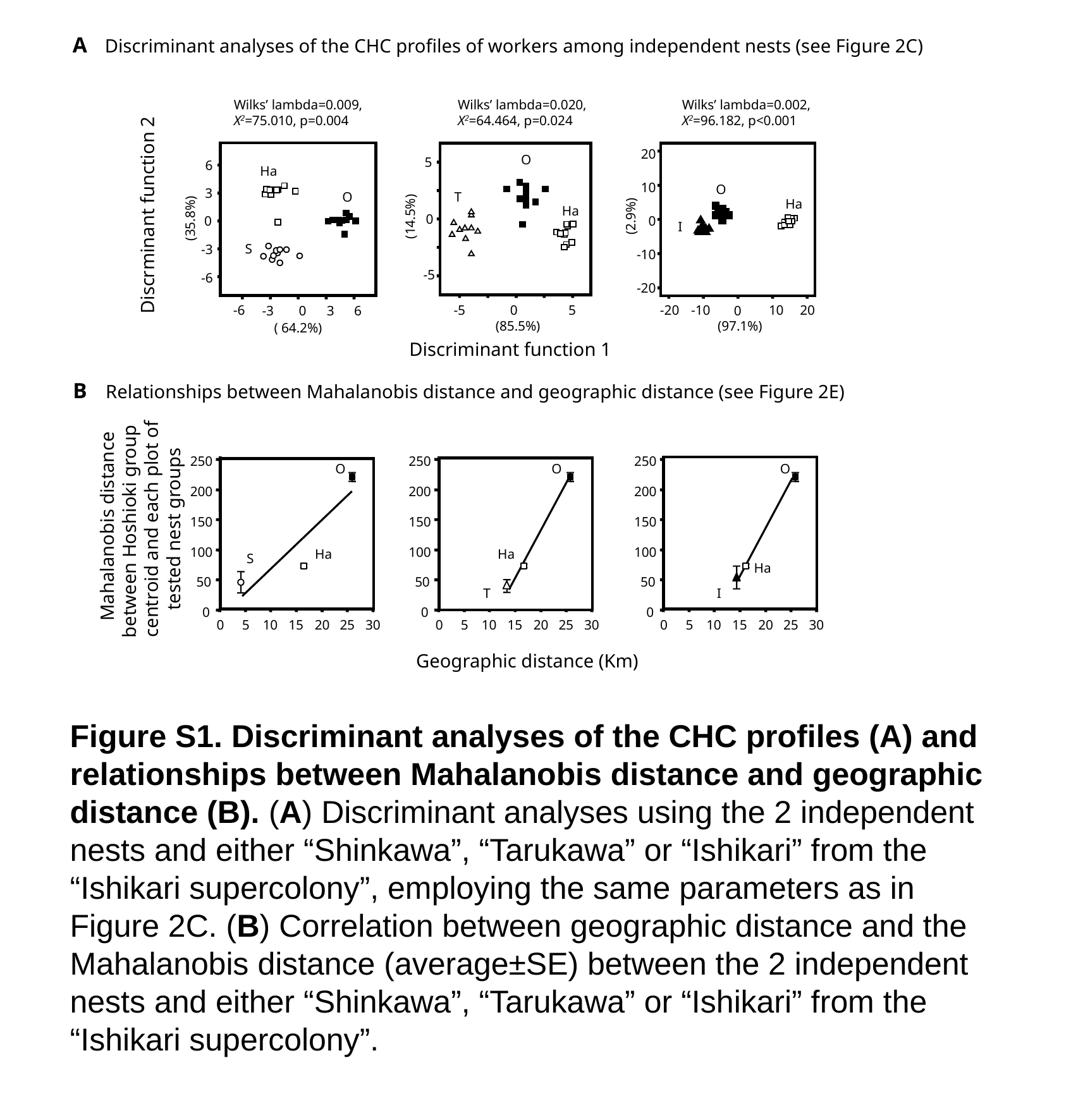

A
 Discriminant analyses of the CHC profiles of workers among independent nests (see Figure 2C)
Wilks’ lambda=0.009, X2=75.010, p=0.004
Wilks’ lambda=0.020, X2=64.464, p=0.024
Wilks’ lambda=0.002, X2=96.182, p<0.001
20
O
5
6
Ha
10
O
3
O
T
Ha
Ha
Discrminant function 2
 (2.9%)
 (14.5%)
(35.8%)
0
0
0
I
S
-3
-10
-5
-6
-20
-6
-5
0
5
-20
-10
10
20
0
-3
0
6
3
 (85.5%)
 (97.1%)
 ( 64.2%)
Discriminant function 1
B
Relationships between Mahalanobis distance and geographic distance (see Figure 2E)
250
200
150
100
50
0
250
200
150
100
50
0
250
200
150
100
50
0
O
O
O
 Mahalanobis distance between Hoshioki group
centroid and each plot of tested nest groups
Ha
Ha
S
Ha
T
I
0
5
10
15
20
25
30
0
5
10
15
20
25
30
0
5
10
15
20
25
30
Geographic distance (Km)
Figure S1. Discriminant analyses of the CHC profiles (A) and relationships between Mahalanobis distance and geographic distance (B). (A) Discriminant analyses using the 2 independent nests and either “Shinkawa”, “Tarukawa” or “Ishikari” from the “Ishikari supercolony”, employing the same parameters as in Figure 2C. (B) Correlation between geographic distance and the Mahalanobis distance (average±SE) between the 2 independent nests and either “Shinkawa”, “Tarukawa” or “Ishikari” from the “Ishikari supercolony”.
