# Supplementary material for: Chemical Discrimination and Aggressiveness via Cuticular Hydrocarbons in a Supercolony-Forming Ant, Formica yessensis
Source: PLoS One. 2012 Oct 24;7(10):e46840. doi: 10.1371/journal.pone.0046840 (PMC3480379; doi:10.1371/journal.pone.0046840)
Supplement: Figure S2 — Comparison of the Mahalanobis distances, based on the discriminant analyses of the CHC profiles. (A) Among the 4 nests within the “Ishikari supercolony”; (B) Among the 3 independent nests: “Hoshioki” within the “Ishikari supercolony”, and “Oshoro” and “Hakkenzan” outside the “Ishikari supercolony”. Each column indicates the average Mahalanobis distance from the nest centroid (indicated by underlined letter) to individual data plots of nests within (white columns) and outside the “Ishikari supercolony” (hatched columns). Single and double asterisks indicate significant difference from the within-nest variation in the Mahalanobis distance of the underlined nests at P<0.05 and <0.001, respectively (F = 11.94 for the graph underlining “Hoshioki”, F = 36.37 for the graph underlining “Shinkawa”, F = 14.52 for the graph underlining “Tarukawa”, F = 33.23 for the graph underlining “Ishikari” in (A); F = 434.39 for the graph underlining “Hoshioki”, F = 175.61 for the graph underlining “Hakkenzan”, F = 390.86 for the graph underlining “Oshoro” in the top panels of (B); F = 288.07 for the graph underlining “Shinkawa”, F = 402.73 for the graph underlining “Hakkenzan”, F = 203.29 for the graph underlining “Oshoro” in the second panels from the top of (B); F = 2070.93 for the graph underlining “Tarukawa”, F = 9805.86 for the graph underlining “Hakkenzan”, F = 6457.37 for the graph underlining “Oshoro” in the third panels from the top of (B); F = 3756.91 for the graph underlining “Ishikari”, F = 5213.13 for the graph underlining “Hakkenzan”, F = 3826.51 for the graph underlining “Oshoro” in the bottom panels of (B); Tukey HSD test). The degrees of freedom are 38 for (A), 28 for the 3 bottom panels of (B), and 29 for other panels in (B). Vertical bars represent the standard errors. (PPT) [file pone.0046840.s002.ppt]

## Slide 1
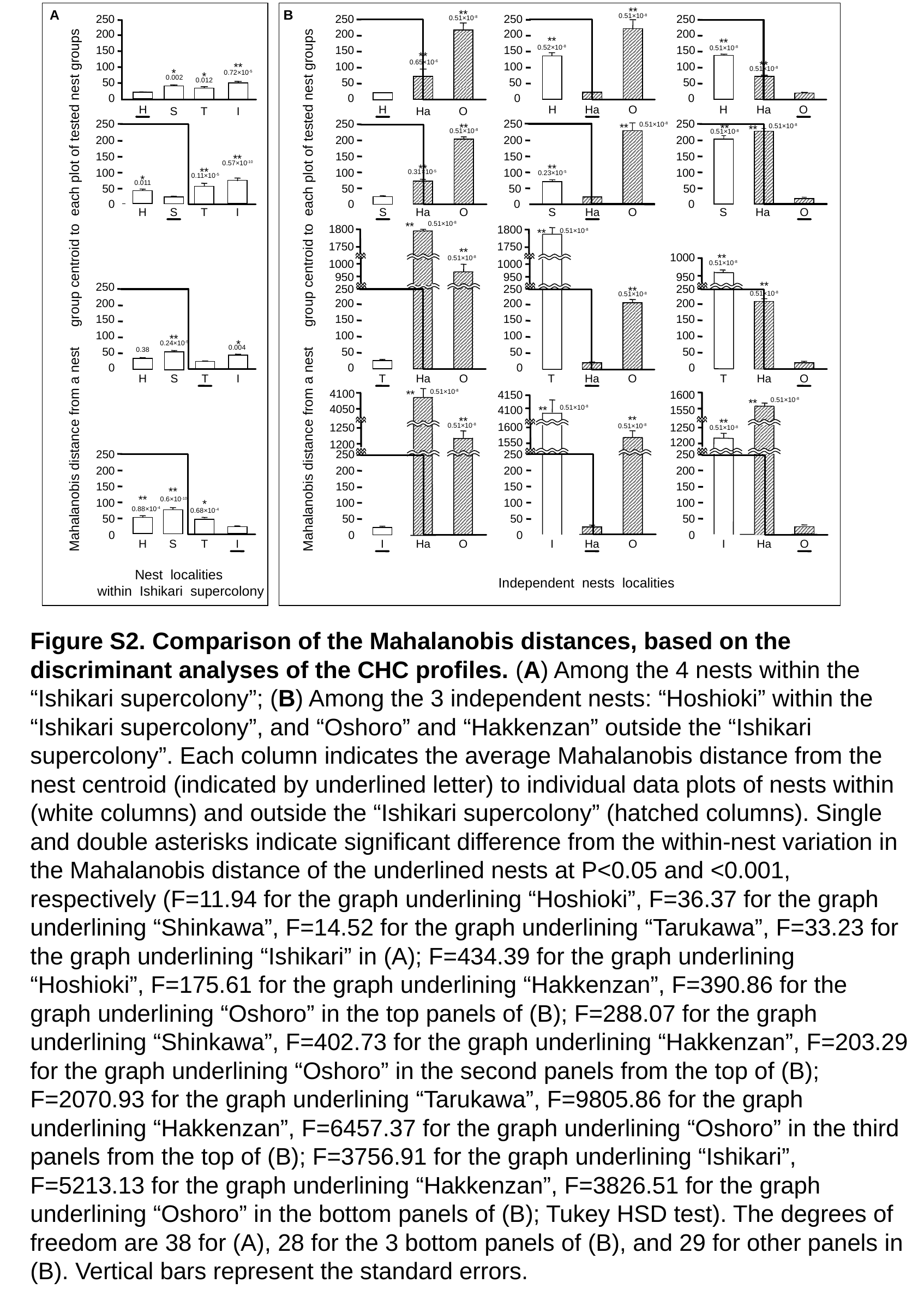

**
A
B
**
0.51×10^-8^
0.51×10^-8^
250
250
250
250
200
200
200
200
**
**
0.52×10^-8^
0.51×10^-8^
150
150
150
150
**
**
0.65×10^-6^
**
100
100
100
100
0.51×10^-8^
*
0.72×10^-5^
*
 0.002
0.012
50
50
50
50
0
0
0
0
H
H
H
Ha
O
H
Ha
O
S
T
I
Ha
O
**
**
0.51×10^-8^
250
250
250
250
**
**
0.51×10^-8^
0.51×10^-8^
0.51×10^-8^
200
200
200
200
**
150
150
150
150
0.57×10^-10^
**
**
**
0.31×10^-5^
0.23×10^-5^
100
100
100
100
 0.11×10^-5^
*
0.011
50
50
50
50
0
0
0
0
H
S
T
I
S
Ha
O
S
Ha
O
S
Ha
O
**
0.51×10^-8^
**
1800
1800
0.51×10^-8^
1750
1750
**
**
0.51×10^-8^
1000
0.51×10^-8^
1000
1000
950
950
950
**
Mahalanobis distance from a nest　group centroid to each plot of tested nest groups
Mahalanobis distance from a nest　group centroid to each plot of tested nest groups
**
250
250
250
250
0.51×10^-8^
0.51×10^-8^
200
200
200
200
150
150
150
150
**
100
100
100
100
*
0.24×10^-5^
0.004
0.38
50
50
50
50
0
0
0
0
H
S
T
I
T
Ha
O
T
Ha
O
T
Ha
O
**
0.51×10^-8^
4100
1600
4150
**
0.51×10^-8^
**
0.51×10^-8^
4050
1550
4100
**
**
**
0.51×10^-8^
0.51×10^-8^
0.51×10^-8^
1600
1250
1250
1550
1200
1200
250
250
250
250
200
200
200
200
150
150
150
150
**
**
0.6×10^-10^
*
100
100
100
100
0.88×10^-4^
0.68×10^-4^
50
50
50
50
0
0
0
0
H
S
T
I
I
Ha
O
I
Ha
O
I
Ha
O
Nest localities
within Ishikari supercolony
Independent nests localities
Figure S2. Comparison of the Mahalanobis distances, based on the discriminant analyses of the CHC profiles. (A) Among the 4 nests within the “Ishikari supercolony”; (B) Among the 3 independent nests: “Hoshioki” within the “Ishikari supercolony”, and “Oshoro” and “Hakkenzan” outside the “Ishikari supercolony”. Each column indicates the average Mahalanobis distance from the nest centroid (indicated by underlined letter) to individual data plots of nests within (white columns) and outside the “Ishikari supercolony” (hatched columns). Single and double asterisks indicate significant difference from the within-nest variation in the Mahalanobis distance of the underlined nests at P<0.05 and <0.001, respectively (F=11.94 for the graph underlining “Hoshioki”, F=36.37 for the graph underlining “Shinkawa”, F=14.52 for the graph underlining “Tarukawa”, F=33.23 for the graph underlining “Ishikari” in (A); F=434.39 for the graph underlining “Hoshioki”, F=175.61 for the graph underlining “Hakkenzan”, F=390.86 for the graph underlining “Oshoro” in the top panels of (B); F=288.07 for the graph underlining “Shinkawa”, F=402.73 for the graph underlining “Hakkenzan”, F=203.29 for the graph underlining “Oshoro” in the second panels from the top of (B); F=2070.93 for the graph underlining “Tarukawa”, F=9805.86 for the graph underlining “Hakkenzan”, F=6457.37 for the graph underlining “Oshoro” in the third panels from the top of (B); F=3756.91 for the graph underlining “Ishikari”, F=5213.13 for the graph underlining “Hakkenzan”, F=3826.51 for the graph underlining “Oshoro” in the bottom panels of (B); Tukey HSD test). The degrees of freedom are 38 for (A), 28 for the 3 bottom panels of (B), and 29 for other panels in (B). Vertical bars represent the standard errors.
