# Supplementary material for: Chemical Discrimination and Aggressiveness via Cuticular Hydrocarbons in a Supercolony-Forming Ant, Formica yessensis
Source: PLoS One. 2012 Oct 24;7(10):e46840. doi: 10.1371/journal.pone.0046840 (PMC3480379; doi:10.1371/journal.pone.0046840)
Supplement: Figure S3 — Raster plots for the experiments presented in Figure 4A and 4B . Impulses of the “Hoshioki” CHC sensilla on the antennae attached to the haeds (A) and “Shinkawa” CHC sensilla on the antennae attached to the haeds (B) and those of the “Hoshioki” CHC sensilla on the antennae detached from the haeds (C), which are sorted into different unit clusters, are plotted against time after beginning of stimulation. H-S-1 and 2, e.g., indicate clusters 1 and 2 corresponding to different shapes of impulse units that were sorted from the impulse train recorded in a “Hoshioki” sensillum stimulated with “Shinkawa” CHCs; H-s-1 to 4, e.g., indicate clusters 1 to 4 corresponding to different shapes of impulse units sorted from the impulse train recorded in the same “Hoshioki” sensillum stimulated with solvent without “Shinkawa” CHCs. Left, Raster plots for the representative electrophysiological responses to CHCs extracted from “Hoshioki”, “Shinkawa”, “Hakkenzan”, and C. japonicus. Right, Raster plots for control recordings of the same sensilla in response to the solvent, 10 mM NaCl plus 0.1% Triton X-100. (PPT) [file pone.0046840.s003.ppt]

## Slide 1
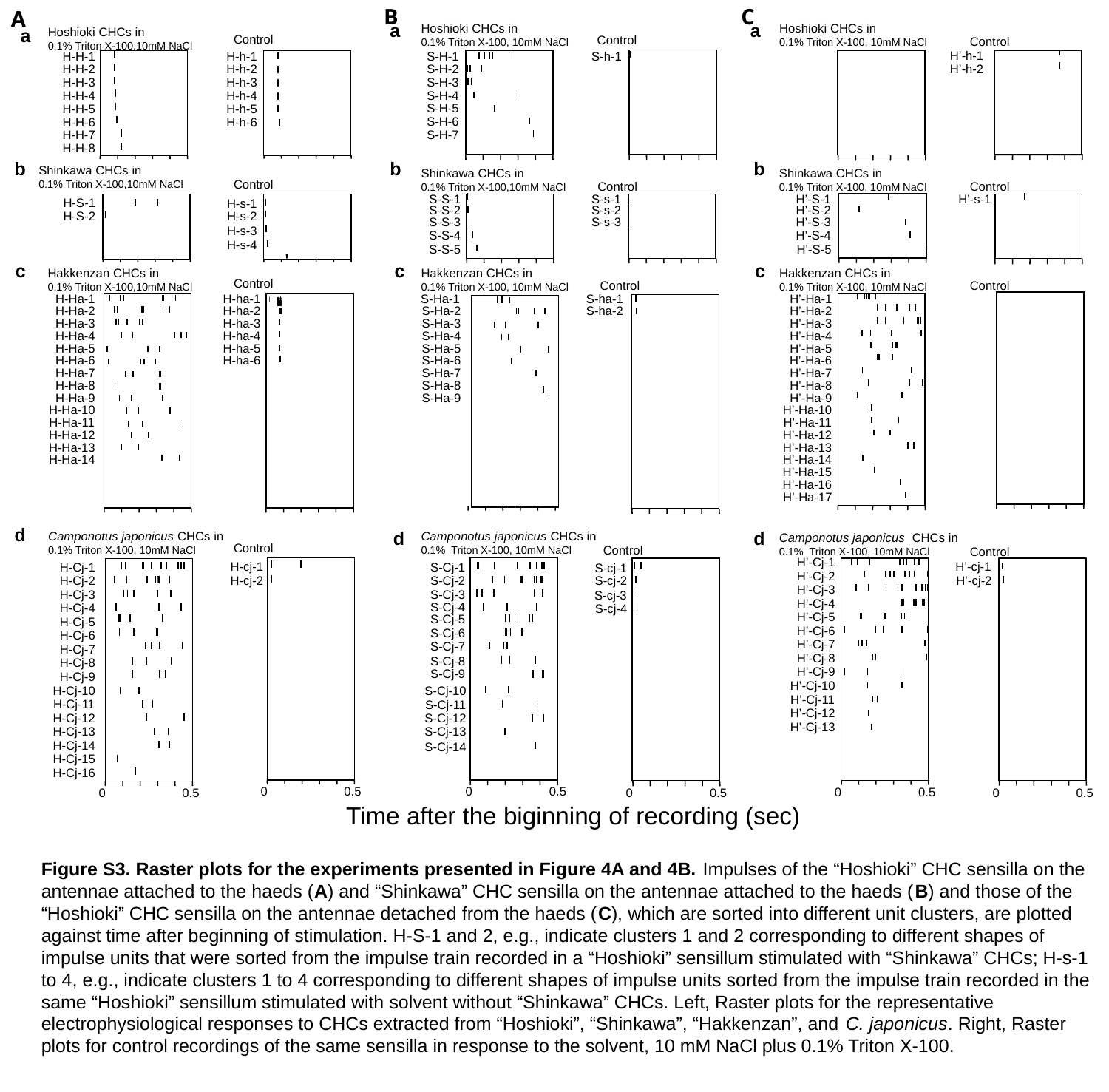

B
C
A
a
a
Hoshioki CHCs in
0.1% Triton X-100, 10mM NaCl
Hoshioki CHCs in
0.1% Triton X-100, 10mM NaCl
a
Hoshioki CHCs in
0.1% Triton X-100,10mM NaCl
Control
Control
Control
H’-h-1
H’-h-2
S-H-1
S-h-1
H-H-1
H-h-1
S-H-2
H-H-2
H-h-2
S-H-3
H-H-3
H-h-3
S-H-4
H-H-4
H-h-4
S-H-5
H-H-5
H-h-5
S-H-6
H-H-6
H-h-6
S-H-7
H-H-7
H-H-8
b
b
b
Shinkawa CHCs in
0.1% Triton X-100,10mM NaCl
Shinkawa CHCs in
0.1% Triton X-100,10mM NaCl
Shinkawa CHCs in
0.1% Triton X-100, 10mM NaCl
Control
Control
Control
S-S-1
S-s-1
H’-S-1
H’-s-1
H-S-1
H-s-1
S-S-2
S-s-2
H’-S-2
H-S-2
H-s-2
S-S-3
S-s-3
H’-S-3
H-s-3
S-S-4
H’-S-4
H-s-4
S-S-5
H’-S-5
c
c
c
Hakkenzan CHCs in
0.1% Triton X-100,10mM NaCl
Hakkenzan CHCs in
0.1% Triton X-100, 10mM NaCl
Hakkenzan CHCs in
0.1% Triton X-100, 10mM NaCl
Control
Control
Control
H-Ha-1
H-ha-1
S-Ha-1
S-ha-1
H’-Ha-1
H-Ha-2
H-ha-2
S-Ha-2
S-ha-2
H’-Ha-2
H-Ha-3
H-ha-3
S-Ha-3
H’-Ha-3
H-Ha-4
H-ha-4
S-Ha-4
H’-Ha-4
H-Ha-5
H-ha-5
S-Ha-5
H’-Ha-5
H-Ha-6
H-ha-6
S-Ha-6
H’-Ha-6
H-Ha-7
S-Ha-7
H’-Ha-7
H-Ha-8
S-Ha-8
H’-Ha-8
H-Ha-9
S-Ha-9
H’-Ha-9
H-Ha-10
H’-Ha-10
H-Ha-11
H’-Ha-11
H-Ha-12
H’-Ha-12
H-Ha-13
H’-Ha-13
H-Ha-14
H’-Ha-14
H’-Ha-15
H’-Ha-16
H’-Ha-17
d
d
d
Camponotus japonicus CHCs in
0.1% Triton X-100, 10mM NaCl
Camponotus japonicus CHCs in
0.1% Triton X-100, 10mM NaCl
Camponotus japonicus CHCs in
0.1% Triton X-100, 10mM NaCl
Control
Control
Control
H’-Cj-1
H-cj-1
H’-cj-1
H-Cj-1
S-Cj-1
S-cj-1
H’-Cj-2
H-Cj-2
H-cj-2
S-Cj-2
H’-cj-2
S-cj-2
H’-Cj-3
H-Cj-3
S-Cj-3
S-cj-3
H’-Cj-4
S-Cj-4
H-Cj-4
S-cj-4
H’-Cj-5
S-Cj-5
H-Cj-5
H’-Cj-6
S-Cj-6
H-Cj-6
H’-Cj-7
S-Cj-7
H-Cj-7
H’-Cj-8
S-Cj-8
H-Cj-8
H’-Cj-9
S-Cj-9
H-Cj-9
H’-Cj-10
H-Cj-10
S-Cj-10
H’-Cj-11
H-Cj-11
S-Cj-11
H’-Cj-12
H-Cj-12
S-Cj-12
H’-Cj-13
H-Cj-13
S-Cj-13
H-Cj-14
S-Cj-14
H-Cj-15
H-Cj-16
0
0.5
0
0.5
0
0.5
0
0.5
0
0.5
0
0.5
Time after the biginning of recording (sec)
Figure S3. Raster plots for the experiments presented in Figure 4A and 4B. Impulses of the “Hoshioki” CHC sensilla on the antennae attached to the haeds (A) and “Shinkawa” CHC sensilla on the antennae attached to the haeds (B) and those of the “Hoshioki” CHC sensilla on the antennae detached from the haeds (C), which are sorted into different unit clusters, are plotted against time after beginning of stimulation. H-S-1 and 2, e.g., indicate clusters 1 and 2 corresponding to different shapes of impulse units that were sorted from the impulse train recorded in a “Hoshioki” sensillum stimulated with “Shinkawa” CHCs; H-s-1 to 4, e.g., indicate clusters 1 to 4 corresponding to different shapes of impulse units sorted from the impulse train recorded in the same “Hoshioki” sensillum stimulated with solvent without “Shinkawa” CHCs. Left, Raster plots for the representative electrophysiological responses to CHCs extracted from “Hoshioki”, “Shinkawa”, “Hakkenzan”, and C. japonicus. Right, Raster plots for control recordings of the same sensilla in response to the solvent, 10 mM NaCl plus 0.1% Triton X-100.
