# Supplementary material for: Chemical Discrimination and Aggressiveness via Cuticular Hydrocarbons in a Supercolony-Forming Ant, Formica yessensis
Source: PLoS One. 2012 Oct 24;7(10):e46840. doi: 10.1371/journal.pone.0046840 (PMC3480379; doi:10.1371/journal.pone.0046840)
Supplement: Figure S4 — Blind experiments on aggressive behavior. (A) Percentage of workers from “Hoshioki” (H), “Shinkawa” (S), “Hakkenzan” (Ha) F. yessensis and C. japonicus (C) (out of total workers tested, respectively) that induced biting behavior of “Hoshioki” (upper plate) and “Shinkawa” workers (lower plate). (B) Percentage of workers from the same nests of F. yessensis and C. japonicus that induced prolonged-antennation behavior of “Hoshioki” (upper plate) and “Shinkawa” workers (lower plate). Resident nests are marked by under bars. Different letters indicate significant differences by Tukey WSD test. (PPT) [file pone.0046840.s004.ppt]

## Slide 1
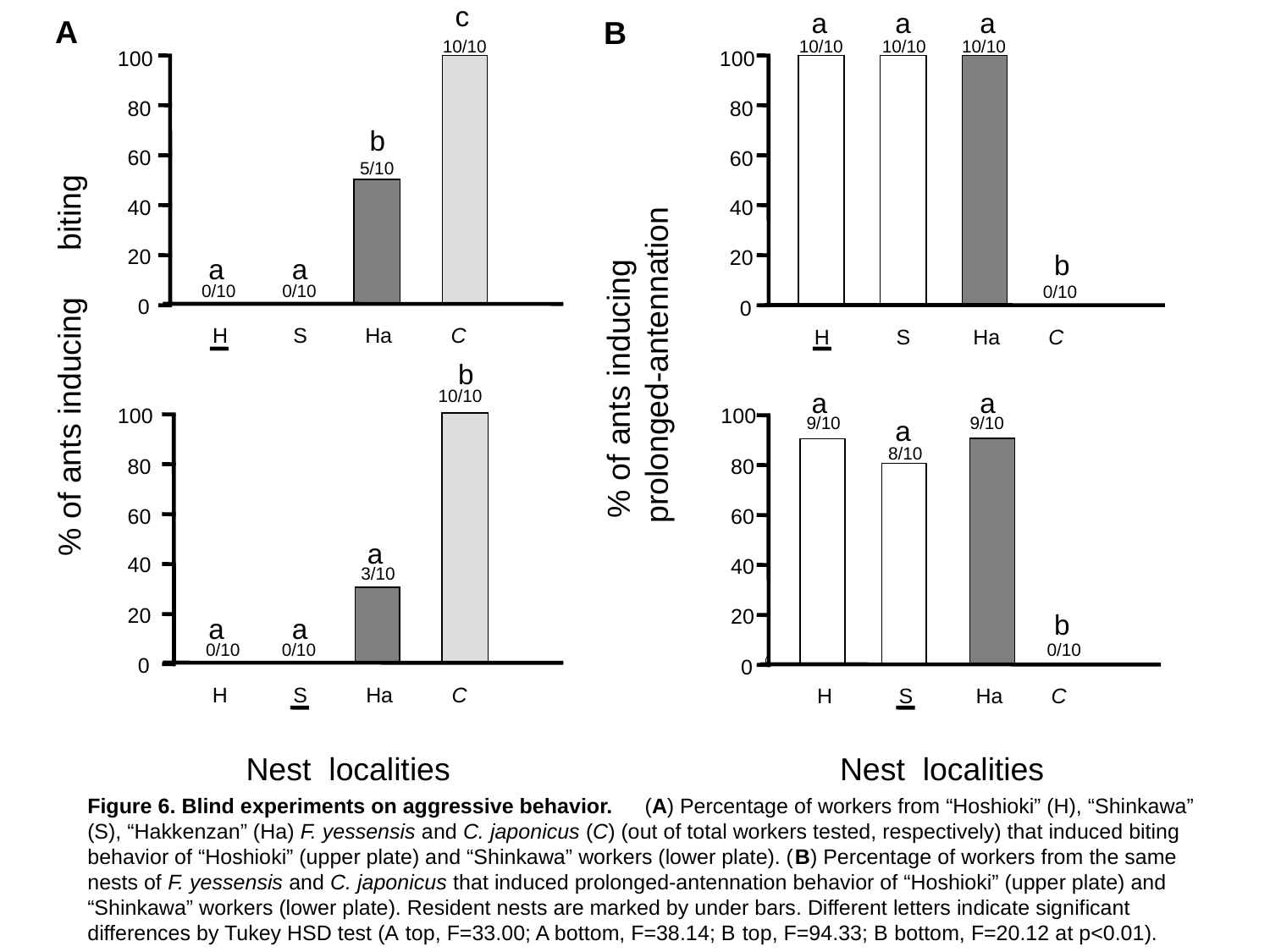

c
a
a
a
A
B
10/10
10/10
10/10
10/10
100
100
80
80
b
60
60
5/10
40
40
b
20
20
a
a
0/10
0/10
0/10
0
0
% of ants inducing　prolonged-antennation
H
S
Ha
C
H
S
Ha
C
% of ants inducing　biting
b
a
a
10/10
100
100
a
9/10
9/10
8/10
80
80
60
60
a
40
40
3/10
b
20
20
a
a
0/10
0/10
0/10
0
0
H
S
Ha
C
H
S
Ha
C
Nest localities
Nest localities
Figure 6. Blind experiments on aggressive behavior.　(A) Percentage of workers from “Hoshioki” (H), “Shinkawa” (S), “Hakkenzan” (Ha) F. yessensis and C. japonicus (C) (out of total workers tested, respectively) that induced biting behavior of “Hoshioki” (upper plate) and “Shinkawa” workers (lower plate). (B) Percentage of workers from the same nests of F. yessensis and C. japonicus that induced prolonged-antennation behavior of “Hoshioki” (upper plate) and “Shinkawa” workers (lower plate). Resident nests are marked by under bars. Different letters indicate significant differences by Tukey HSD test (A top, F=33.00; A bottom, F=38.14; B top, F=94.33; B bottom, F=20.12 at p<0.01).
