# Supplementary material for: Chemical Discrimination and Aggressiveness via Cuticular Hydrocarbons in a Supercolony-Forming Ant, Formica yessensis
Source: PLoS One. 2012 Oct 24;7(10):e46840. doi: 10.1371/journal.pone.0046840 (PMC3480379; doi:10.1371/journal.pone.0046840)
Supplement: Figure S5 — Correlates of biting behaviors, Mahalanobis distances of CHC profiles, and genetic relatedness in resident or invader ants. (A) Correlation between % of invader ants and of glass beads inducing biting behavior of resident “Hoshioki” ants. (B) Correlation between aggressive behavior and average of Mahalanobis distances of CHC profiles between resident “Hoshioki” ants and invader ants. (C) Same as (B) but % of glass beads inducing biting instead of % of ants inducing biting. (D) Correlation between chemical similarity (Mahalanobis distances of CHC profiles) and genetic relatedness between resident and invader ants. (E) Correlation between genetic relatedness and aggressive behavior in encounters involving resident “Hoshioki” and invader ants. (F) Same as (E) but % of glass beads inducing biting instead of % of ants inducing biting. Indicated r-values are correlation coefficients. The straight lines are regression lines drawn by the least-squares method. (PPT) [file pone.0046840.s005.ppt]

## Slide 1
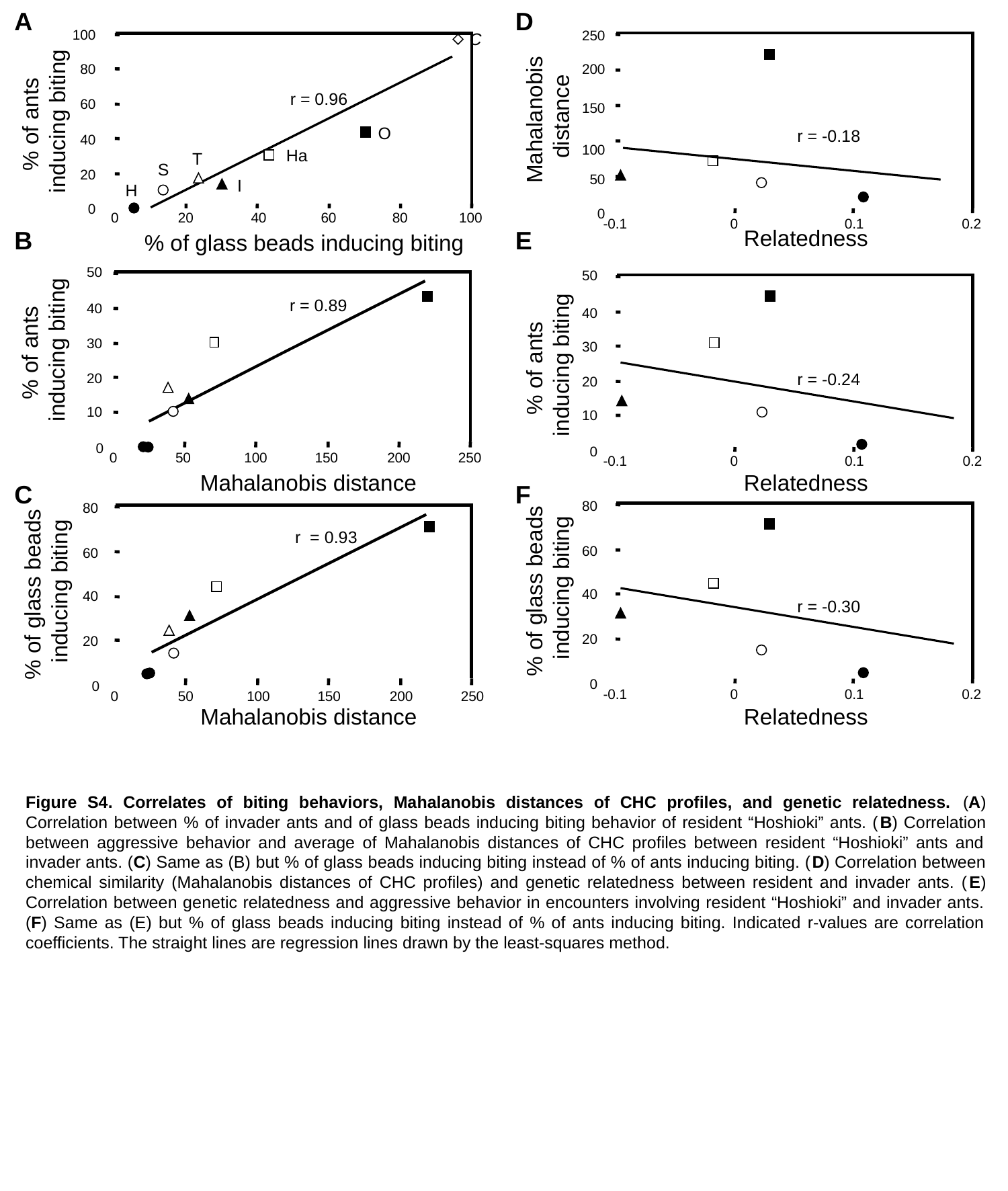

A
D
100
250
C
80
200
 r = 0.96
% of ants
 inducing biting
Mahalanobis
 distance
60
150
r = -0.18
O
40
100
Ha
T
S
20
50
I
H
0
0
0
20
40
60
80
100
-0.1
0
0.1
0.2
B
E
% of glass beads inducing biting
Relatedness
50
50
 r = 0.89
40
40
% of ants
 inducing biting
30
30
% of ants
inducing biting
r = -0.24
20
20
10
10
0
0
0
50
100
150
200
250
-0.1
0
0.1
0.2
Mahalanobis distance
Relatedness
C
F
80
80
r = 0.93
60
60
% of glass beads
 inducing biting
% of glass beads
 inducing biting
40
40
r = -0.30
20
20
0
0
-0.1
0
0.1
0.2
0
50
100
150
200
250
Mahalanobis distance
Relatedness
Figure S4. Correlates of biting behaviors, Mahalanobis distances of CHC profiles, and genetic relatedness. (A) Correlation between % of invader ants and of glass beads inducing biting behavior of resident “Hoshioki” ants. (B) Correlation between aggressive behavior and average of Mahalanobis distances of CHC profiles between resident “Hoshioki” ants and invader ants. (C) Same as (B) but % of glass beads inducing biting instead of % of ants inducing biting. (D) Correlation between chemical similarity (Mahalanobis distances of CHC profiles) and genetic relatedness between resident and invader ants. (E) Correlation between genetic relatedness and aggressive behavior in encounters involving resident “Hoshioki” and invader ants. (F) Same as (E) but % of glass beads inducing biting instead of % of ants inducing biting. Indicated r-values are correlation coefficients. The straight lines are regression lines drawn by the least-squares method.
