# Supplementary material for: Chemical Discrimination and Aggressiveness via Cuticular Hydrocarbons in a Supercolony-Forming Ant, Formica yessensis
Source: PLoS One. 2012 Oct 24;7(10):e46840. doi: 10.1371/journal.pone.0046840 (PMC3480379; doi:10.1371/journal.pone.0046840)
Supplement: Figure S6 — Coleman rarefaction curve for “Hoshioki” workers. For the analysis of genetic structure, 48 ant individuals were used. The total number of alleles at seven microsatellite loci was counted by randomly choosing each number of individuals (n) from the 48 ants, and the mean number of alleles with standard deviation (bar) was calculated from 1,000 replications. The curve reaches plateau around n = 30, indicating that the sample size 48 is large enough for the analysis of genetic structure. In all five nests, the Coleman rarefaction curve reached plateau at n = 30–35 (data not shown). (PPT) [file pone.0046840.s006.ppt]

## Slide 1
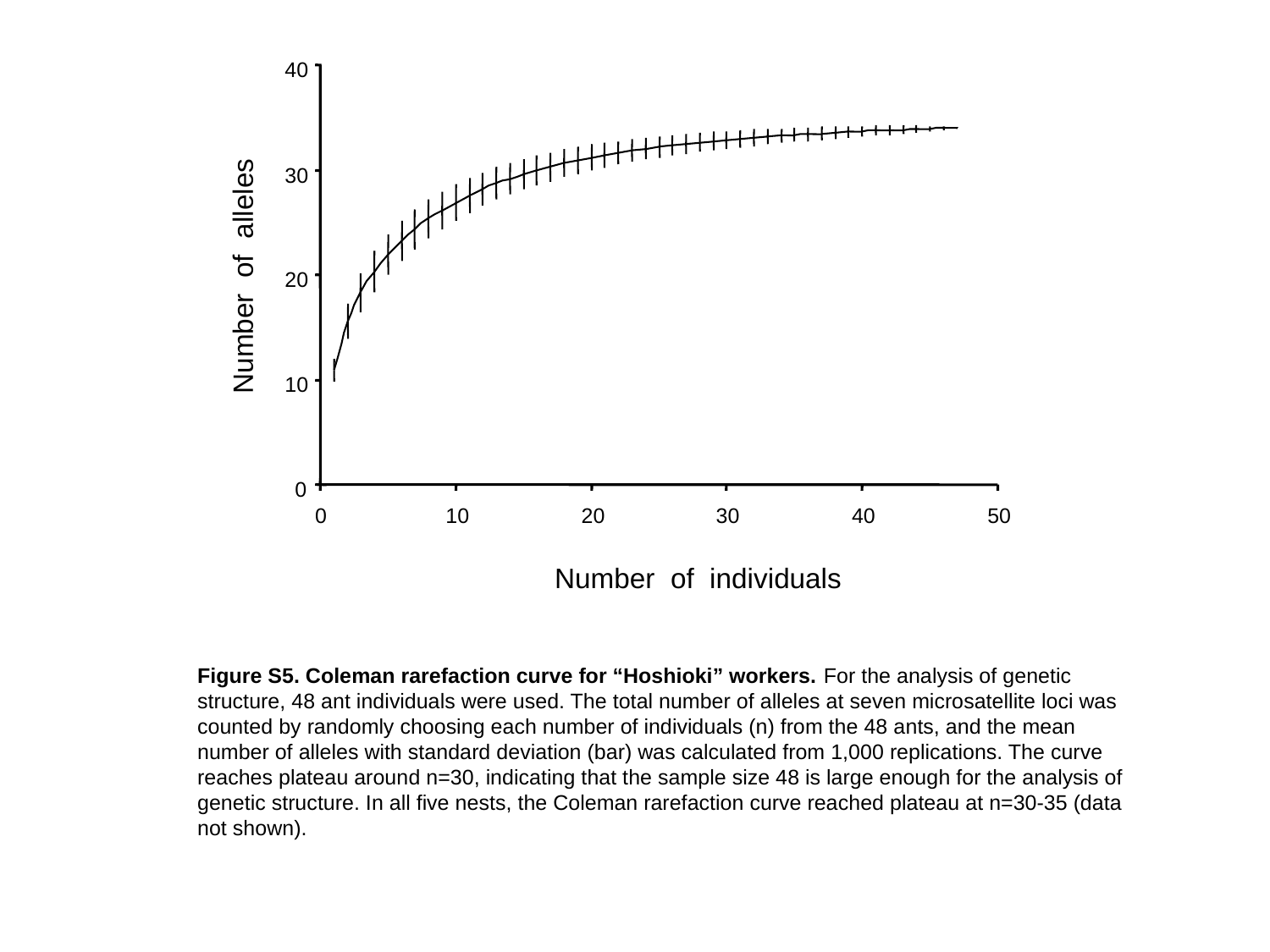

40
30
20
10
0
0
10
20
30
40
50
Number of alleles
Number of individuals
Figure S5. Coleman rarefaction curve for “Hoshioki” workers. For the analysis of genetic structure, 48 ant individuals were used. The total number of alleles at seven microsatellite loci was counted by randomly choosing each number of individuals (n) from the 48 ants, and the mean number of alleles with standard deviation (bar) was calculated from 1,000 replications. The curve reaches plateau around n=30, indicating that the sample size 48 is large enough for the analysis of genetic structure. In all five nests, the Coleman rarefaction curve reached plateau at n=30-35 (data not shown).
