# Supplementary material for: Chemical Discrimination and Aggressiveness via Cuticular Hydrocarbons in a Supercolony-Forming Ant, Formica yessensis
Source: PLoS One. 2012 Oct 24;7(10):e46840. doi: 10.1371/journal.pone.0046840 (PMC3480379; doi:10.1371/journal.pone.0046840)
Supplement: Table S1 — Average number of sensilla basiconica (CHC sensilla) in a worker’s antenna. (PPT) [file pone.0046840.s007.ppt]

## Slide 1
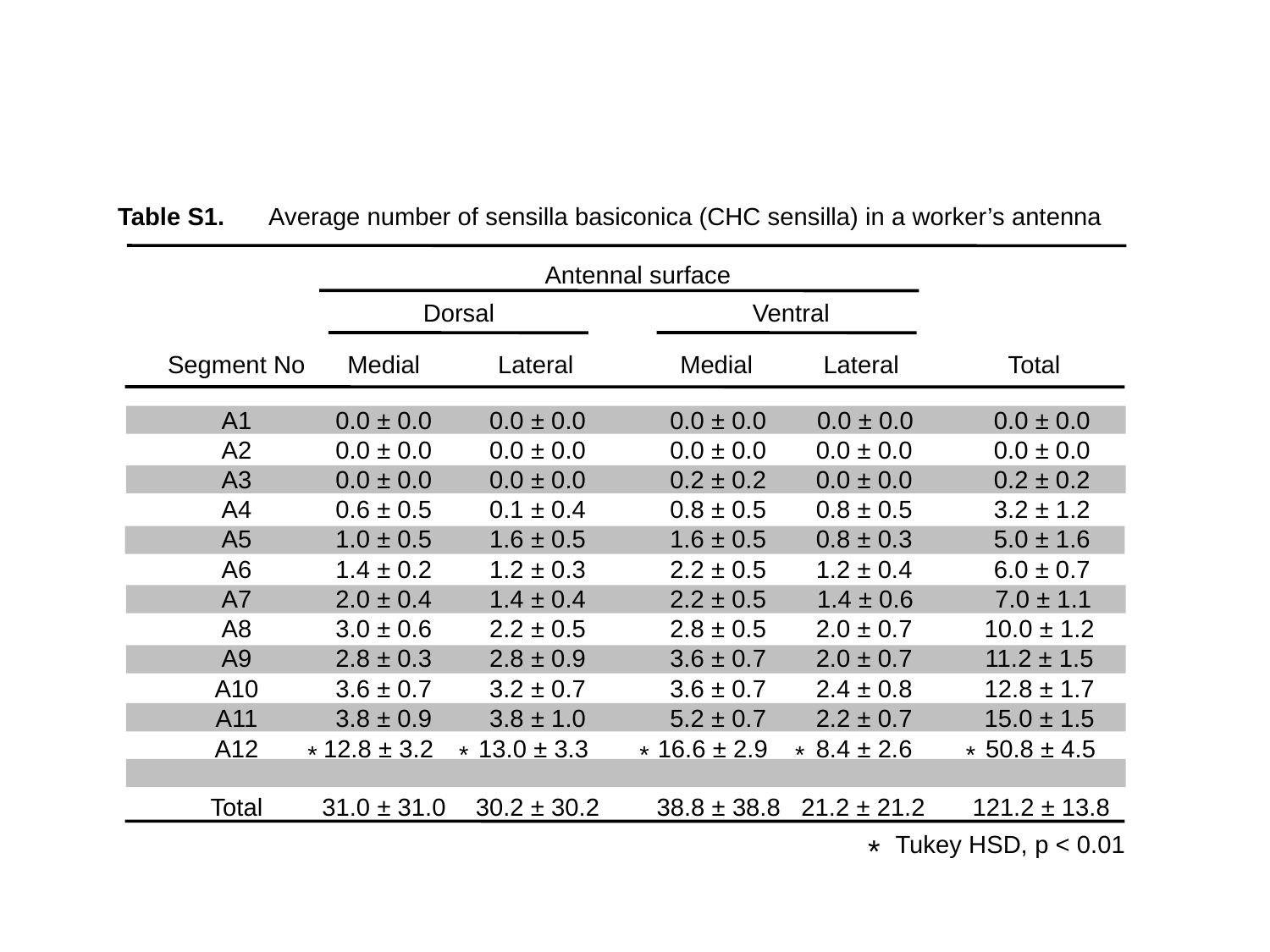

Table S1. 　Average number of sensilla basiconica (CHC sensilla) in a worker’s antenna
Antennal surface
Dorsal
Ventral
Segment No
Medial
Lateral
Medial
Lateral
Total
A1
0.0 ± 0.0
0.0 ± 0.0
0.0 ± 0.0
0.0 ± 0.0
0.0 ± 0.0
A2
0.0 ± 0.0
0.0 ± 0.0
0.0 ± 0.0
0.0 ± 0.0
0.0 ± 0.0
A3
0.0 ± 0.0
0.0 ± 0.0
0.2 ± 0.2
0.0 ± 0.0
0.2 ± 0.2
A4
0.6 ± 0.5
0.1 ± 0.4
0.8 ± 0.5
0.8 ± 0.5
3.2 ± 1.2
A5
1.0 ± 0.5
1.6 ± 0.5
1.6 ± 0.5
0.8 ± 0.3
5.0 ± 1.6
A6
1.4 ± 0.2
1.2 ± 0.3
2.2 ± 0.5
1.2 ± 0.4
6.0 ± 0.7
A7
2.0 ± 0.4
1.4 ± 0.4
2.2 ± 0.5
1.4 ± 0.6
7.0 ± 1.1
A8
3.0 ± 0.6
2.2 ± 0.5
2.8 ± 0.5
2.0 ± 0.7
10.0 ± 1.2
A9
2.8 ± 0.3
2.8 ± 0.9
3.6 ± 0.7
2.0 ± 0.7
11.2 ± 1.5
A10
3.6 ± 0.7
3.2 ± 0.7
3.6 ± 0.7
2.4 ± 0.8
12.8 ± 1.7
A11
3.8 ± 0.9
3.8 ± 1.0
5.2 ± 0.7
2.2 ± 0.7
15.0 ± 1.5
*
*
*
*
*
A12
12.8 ± 3.2
13.0 ± 3.3
16.6 ± 2.9
8.4 ± 2.6
50.8 ± 4.5
Total
31.0 ± 31.0
30.2 ± 30.2
38.8 ± 38.8
21.2 ± 21.2
121.2 ± 13.8
 Tukey HSD, p < 0.01
*
